# The Crosswise Model for Surveys on Sensitive Topics: A General Framework for Item Selection and Statistical Analysis

**DOI:** 10.1007/s11336-024-09976-3

**Published:** 2024-05-28

**Authors:** Marco Gregori, Martijn G. De Jong, Rik Pieters

**Affiliations:** 1 Department of Marketing (Room 3.201), Warwick Business School, University of Warwick, Scarman Road, Coventry, CV4 7AL UK; 2 Erasmus University Rotterdam, Rotterdam, The Netherlands; 3 Tilburg University, Tilburg, The Netherlands

**Keywords:** surveys, sensitive questions, truth-telling techniques, Bayesian statistics, item response theory

## Abstract

When surveys contain direct questions about sensitive topics, participants may not provide their true answers. Indirect question techniques incentivize truthful answers by concealing participants’ responses in various ways. The Crosswise Model aims to do this by pairing a sensitive target item with a non-sensitive baseline item, and only asking participants to indicate whether their responses to the two items are the same or different. Selection of the baseline item is crucial to guarantee participants’ perceived and actual privacy and to enable reliable estimates of the sensitive trait. This research makes the following contributions. First, it describes an integrated methodology to select the baseline item, based on conceptual and statistical considerations. The resulting methodology distinguishes four statistical models. Second, it proposes novel Bayesian estimation methods to implement these models. Third, it shows that the new models introduced here improve efficiency over common applications of the Crosswise Model and may relax the required statistical assumptions. These three contributions facilitate applying the methodology in a variety of settings. An empirical application on attitudes toward LGBT issues shows the potential of the Crosswise Model. An interactive app, Python and MATLAB codes support broader adoption of the model.

**Supplementary Information:**

The online version contains supplementary material available at 10.1007/s11336-024-09976-3.

Policy makers and researchers in psychology, sociology, business and economics often have a keen interest in understanding the public’s behaviors, beliefs and attitudes with respect to sensitive topics. Efforts to ensure truthful responses to sensitive survey questions have led to the development of indirect question techniques (Lensvelt-Mulders et al., 2005; Blair et al., 2020; Tourangeau and Yan, 2007). These indirect techniques “conceal” in various ways the true response of survey participants to a sensitive question to protect their privacy and to induce honest answers. Randomized response techniques (RRTs, introduced by Warner (1965)) add random variation to the survey participants’ true answer (Blair et al., 2015; Lensvelt-Mulders et al., 2005). List Experiments (Blair et al., 2020; Kuklinski et al., 1997) ask survey participants to respond to a list of statements, including a sensitive item, and to indicate only how many items in the list they affirm.

The Crosswise Model (CM) methodology is a recent alternative (Yu et al., 2008) with potentially important advantages over the more commonly known methodologies^1^. It provides survey participants with a pair of items: one sensitive, target item and one non-sensitive, baseline item. It merely asks participants to indicate whether their answers to the target and the baseline item in the pair are the same or different. Thus, analysts do not observe the true answer of survey participants to the target item: it is “concealed” to protect the survey participants’ privacy. The aim of the CM methodology is to estimate the prevalence of the target item and relate the hidden answers to the target item to other variables of interest, such as socio-demographic, psychographic and behavioral variables. Recent reviews (Sagoe et al., 2021; Schnell and Thomas, 2021) counted 45 applications of the CM, indicating its growing popularity. Existing applications of the CM methodology typically rest on the assumption that the prevalence of the baseline item in the sample is known (Yu et al., 2008; Schnell and Thomas, 2021; Sagoe et al., 2021). For instance, a commonly used baseline item is “I was born in January or February”: the probability of the event is typically assumed to be approximately 1/6 (Höglinger and Diekmann, 2017; Höglinger and Jann, 2018). Under this assumption, the aggregate prevalence of the sensitive trait can be readily derived from the crosswise responses.

The CM methodology has several potential advantages over other indirect question techniques. First, the instructions are easy to understand and do not require an understanding of probability and the availability of a trustworthy randomization device, as with the RRTs (Hoffmann et al., 2017; John et al., 2018; Chuang et al., 2021). Second, it uses only a single baseline item to ask the target item, which is more efficient than List Experiments. Third, the CM guarantees full privacy protection to the survey participant: either answer (same or different) does not convey whether the participant affirms or denies the sensitive target item. In contrast, in both RRTs and List Experiments survey participants may prefer to select answers, implying that they do not affirm the sensitive item (Atsusaka and Stevenson, 2021; Blair and Imai, 2012; Nepusz et al., 2014; Wolter and Preisendörfer, 2013).

Despite its promise, existing research has also indicated weaknesses of the CM methodology. As with other indirect question techniques, implementing the CM requires large sample sizes, as it increases the estimated variance of the target item compared to direct questioning (Qiu et al., 2022; Reiber et al., 2020). Focusing on sample subsets—such as by gender or age—and some extensions of the CM to correct for random responses result in even lower efficiency (Atsusaka and Stevenson, 2021). Second, presenting the baseline item within the CM should not affect participants’ answers: for instance, survey participants should respond to the baseline item similarly, regardless of whether it is asked directly or in the CM. If this “response invariance” assumption (De Jong and Pieters, 2019) is violated, the assumed prevalence of the baseline item is incorrect and the estimated prevalence of the target item will be biased. Third and finally, to ensure their cooperation, survey participants must perceive both the CM instructions and the baseline item as guaranteeing the stated privacy protection (Hoffmann et al., 2017; Jerke et al., 2019). Awkward or strange baseline items might compromise this. Existing applications of the CM methodology have predominantly (78 % out of 45 studies in the meta-analysis by Sagoe et al. (2021)) used a baseline item concerning day or month of birth of the participant. Such baseline items in the CM pose several conceptual and practical problems, suggesting that the usage of alternative baseline items may be warranted (Sayed et al., 2022). These issues may prevent correct estimation of the prevalence of the sensitive trait in applications of the CM.

Here we develop novel statistical models that can improve efficiency, relax assumptions of the CM methodology and allow more variety in the set of baseline items. The proposed models rely on Item Response Theory (IRT) and on modeling the binary responses to predict the baseline item (De Jong and Pieters, 2019; Kuha and Jackson, 2014). Usage of alternative baseline items, however, presents significant challenges related to data collection and modeling. Beyond the issues already discussed, indirect question techniques using baseline items that do not rely on randomization—such as the List Experiment—typically assume that these items are statistically independent of the target item of interest. While this assumption is usually untestable, IRT modeling of the baseline item as proposed by De Jong and Pieters (2019) allows to detect and model dependence between the baseline and target item. We develop statistical tests to explicitly check for statistical independence as well as modeling dependence between the baseline and target item in the CM. Data collection requirements for correct model estimation are addressed as well.

More generally, we develop an integrated methodology for item selection and address statistical issues with respect to four models. Models CM1 and CM2 have been used in existing literature for data analysis with the CM. Models CM3 and CM4 are new and introduced here. All statistical models are suitable for individual-level inference, which allows analysts to relate background variables to the hidden response to the sensitive target item. The resulting taxonomy is, to the best of our knowledge, the first systematic organization of possible approaches to select the baseline item. Importantly, different statistical models can be estimated with the same baseline item. This systematic approach will help researchers select and evaluate suitable baseline items, understand model assumptions and analyze data. An empirical application on attitudes toward LGBT issues illustrates the usefulness of the proposed methodology.

All models are estimated using Bayesian methodology. Both MATLAB and Python codes are added to replicate the empirical application. We also include an interactive app to facilitate implementation of the proposed statistical models for the CM. The supplemental material includes instructions for the use of the app.

## Variants of the Crosswise Model

Regardless of the choice of baseline item and statistical model, the CM presents the following statistical structure. The probability that participant *i* answers the target item $U_i$ affirmatively is denoted ${{\,\mathrm{\mathbb {P}}\,}}(U_{i} = 1)$, and the probability that s/he answers the baseline item $Z_i$ affirmatively is denoted ${{\,\mathrm{\mathbb {P}}\,}}(Z_{i} = 1)$. The probability of observing the answer “My response to both items is different”, that is, “one yes and one no”, denoted as $Y_i = 1$, is then:

1 $$\begin{aligned} {\begin{matrix} {{\,\mathrm{\mathbb {P}}\,}}(Y_i = 1) &{}= {{\,\mathrm{\mathbb {P}}\,}}(U_{i} = 1, Z_{i} = 0)+{{\,\mathrm{\mathbb {P}}\,}}(U_{i} = 0, Z_{i} = 1) \\ {} &{}= {{\,\mathrm{\mathbb {P}}\,}}(U_{i} = 1 | Z_{i} = 0) {{\,\mathrm{\mathbb {P}}\,}}(Z_{i} = 0)+{{\,\mathrm{\mathbb {P}}\,}}(U_{i} = 0 | Z_{i} = 1) {{\,\mathrm{\mathbb {P}}\,}}(Z_{i} = 1) \end{matrix}} \end{aligned}$$

The probability of observing the answer “My response to both items is the same”, that is, “both yes or both no”, denoted as $Y_i = 0$, is then:

$$\begin{aligned} {{\,\mathrm{\mathbb {P}}\,}}(Y_i = 0) = {{\,\mathrm{\mathbb {P}}\,}}(U_{i} = 0 | Z_{i} = 0) {{\,\mathrm{\mathbb {P}}\,}}(Z_{i} = 0)+{{\,\mathrm{\mathbb {P}}\,}}(U_{i} = 1 | Z_{i} = 1) {{\,\mathrm{\mathbb {P}}\,}}(Z_{i} = 1) \end{aligned}$$

Notice that only the response $Y_i$ is observed. The aim is to infer ${{\,\mathrm{\mathbb {P}}\,}}(U_{i} = 1)$, that is, the probability of affirming the target item, based on Eq. 1. To identify the unknown (namely, ${{\,\mathrm{\mathbb {P}}\,}}(U_{i} = 1)$) we need an estimate of the probability of affirming the baseline item ${{\,\mathrm{\mathbb {P}}\,}}(Z_{i} = 1)$. Estimation of this probability depends on the specific baseline item selected and the statistical model of choice. Table 1 summarizes the possible variants of the CM- based on the data collection (type of baseline item in Column 3) and the statistical model (Columns 4 and 5). Table 2 provides an overview of pros and cons

**Table 1:** Inferring response to the sensitive target item: four possible models for choice and analysis of baseline item.

Crosswise	1. Response to baseline item	2. Item type	3. Estimation	4. Model
model			method	equation
CM1	Between-groups(probability known)	a. Outcome of randomization device	Beta-binomial model	$\delta$ (known)
		b. Item with known probability		
CM2	Between-groups	Any item		$\delta$ (estimated)
	(probability estimated)			
CM3	Within-participant	a. Validated multi-item	a. IRT-based model	a. $\Phi (\alpha _{bas} \theta _{i} - \gamma _{bas})$
		scale with known reliability		
		b. Any item	b. Binary Response	b. $g(\delta _0 + \textbf{x}_i^\intercal \varvec{\delta }_{bas})$
			model (e.g., probit)	
CM4	Hybrid: probability estimated within-participant	a. Validated multi-item	a. IRT-Based model	a. $\Phi (\alpha _{bas} \theta _{i} - \gamma _{bas})$
		scale with known reliability		
	and between-groups	b. Any item	b. Binary Response	b. $g(\delta _0 + \textbf{x}_i^\intercal \varvec{\delta }_{bas})$
			model (e.g., probit)

**Table 2:** Pros and cons of respective models.

Model	Pros	Cons
Model CM1a	Analysis model is straightforward	Item instructions require use of randomizer
Model CM1b	Analysis model is straightforward	1. Item instructions may be salient/weird
		2. Item can have potentially high efficiency, but survey
		participant must believe it is sufficiently rare/protected
Model CM2	Analysis model is straightforward	1. Low efficiency/requires separate control group
		2. Assumption of response invariance
Model CM3	1. Does not assume response invariance	Requires administering outside-the-CM items
	2. Allows testing/modeling dependence	
Model CM4	1.High efficiency	1. Requires separate control group and outside-the-CM items
	2. Allows testing/modeling dependence	2. Assumption of response invariance

### Statistical Model CM1: Known Baseline Prevalence

#### Model CM1a: Randomization Device

In model CM1a, the baseline item in the data collection concerns the outcome of a randomization device available to the survey participant, as typically done with RRTs (Mirzazadeh et al., 2018). For instance, the baseline item may be whether “the outcome of a die roll (available to survey participants) is a 2”. Hence, the responses to the two statements in the CM are conditionally independent, i.e. ${{\,\mathrm{\mathbb {P}}\,}}(U_{i} = 1 | Z_{i} ) = {{\,\mathrm{\mathbb {P}}\,}}(U_{i} = 1 )$ in Eq. 1. Thus,

$$\begin{aligned} {{\,\mathrm{\mathbb {P}}\,}}(Y_i = 1)&= {{\,\mathrm{\mathbb {P}}\,}}(U_{i} = 1 ) {{\,\mathrm{\mathbb {P}}\,}}(Z_{i} = 0)+{{\,\mathrm{\mathbb {P}}\,}}(U_{i} = 0 ) {{\,\mathrm{\mathbb {P}}\,}}(Z_{i} = 1) \\ {{\,\mathrm{\mathbb {P}}\,}}(Y_i = 0)&= {{\,\mathrm{\mathbb {P}}\,}}(U_{i} = 1 ) {{\,\mathrm{\mathbb {P}}\,}}(Z_{i} = 1)+{{\,\mathrm{\mathbb {P}}\,}}(U_{i} = 0 ) {{\,\mathrm{\mathbb {P}}\,}}(Z_{i} = 0) \end{aligned}$$

Given prevalence of the target item $\pi = {{\,\mathrm{\mathbb {P}}\,}}(U_i=1)$, the likelihood for individual response $Y_i$ is:

2 $$\begin{aligned} \mathcal {L}(Y_i|\pi , \delta ) = {{\,\mathrm{\mathbb {P}}\,}}(Y_i = 1|\pi , \delta )^{Y_i} \times (1-{{\,\mathrm{\mathbb {P}}\,}}(Y_i = 1|\pi , \delta ) )^{1-Y_i} \end{aligned}$$

where the prevalence of the baseline item ${{\,\mathrm{\mathbb {P}}\,}}(Z_{i} = 1) = \delta$ is the probability of the specified outcome of the randomization device. Then, $\delta$ is a fixed parameter known to the analyst. For instance, in the example given above $\delta =1/6$. The probability of having the sensitive target item can be computed as follows:

3 $$\begin{aligned} \pi = {{\,\mathrm{\mathbb {P}}\,}}(U_{i} = 1) = \frac{{{\,\mathrm{\mathbb {P}}\,}}(Y_i = 1) - {{\,\mathrm{\mathbb {P}}\,}}(Z_{i} = 1)}{1 - 2 {{\,\mathrm{\mathbb {P}}\,}}(Z_{i} = 1)} = \frac{{{\,\mathrm{\mathbb {P}}\,}}(Y_i = 1) - \delta }{1 - 2 \delta } \end{aligned}$$

The probability of affirming the sensitive item can then be estimated. A caveat is that the target response is not identified if ${{\,\mathrm{\mathbb {P}}\,}}(Z_{i} = 1) = \delta = 50 \%$ (under Eq. 3). Additionally, analysts are often interested in relating the sensitive target item to observed covariates denoted as $\textbf{x}_i$, that is, they are interested in ${{\,\mathrm{\mathbb {P}}\,}}(U_{i} = 1| \textbf{x}_i)$. The probability of affirming the target item then varies with the survey participant’s characteristics. Using a probit link function, where $\Phi (\cdot )$ is the normal cumulative distribution function, this becomes:

4 $$\begin{aligned} {{\,\mathrm{\mathbb {P}}\,}}(U_{i} = 1| \textbf{x}_i, \varvec{\beta }) = \Phi (\beta _0 + \textbf{x}_i^\intercal \varvec{\beta }_X) \end{aligned}$$

Past research has documented various downsides of using randomization devices to protect privacy, such as trust and understanding issues (Landsheer et al., 1999; John et al., 2018; Wolter and Preisendörfer, 2013). This motivates model CM1b, using baseline items not involving a randomization device.

#### Model CM1b: Assumed Prevalence

Model CM1b is used, to the best of our knowledge, in almost all applications of the CM (Sagoe et al., 2021; Schnell and Thomas, 2021). Typical baseline items for data collection of model CM1b are “My mother’s birthday is in January or February” or “My address number begins with 6”. The responses to these baseline items aim to be equivalent to the outcomes of a randomization device. Thus, although this approach is statistically equivalent to model CM1a, the implementation differs.

Model CM1b is straightforward, since the probability of affirming the item can be treated as known. However, the model presents several limitations (Sayed et al., 2022). First, if survey participants do not perceive items such as one’s day or month of birth, or number of the street address as privacy protecting, then they might not comply with the instructions. For instance, they might worry that the analyst might have access to this, for instance, via web-scraping or earlier waves in case the study is part of longitudinal research (Jerke et al., 2019). This can be prevented by asking impersonal items like “An acquaintance’s birthday is in January or February” (Höglinger and Jann, 2018; Jann et al., 2011). However, these baseline items might appear weird (Kuha and Jackson, 2014), thus increasing participants’ likelihood of answering randomly and thereby the measurement error of the baseline item (Höglinger and Diekmann, 2017). Second, researchers may often be interested in using more than one CM question, for instance when they want to collect data on multiple sensitive issues or when using an anchoring item to correct for non-compliance (Sayed et al., 2022; Atsusaka and Stevenson, 2021). If multiple CMs are administered repeatedly with similar baseline items, as is common (Höglinger and Jann, 2018; Roberts and John, 2014)), participants might worry that analysts can deduce the answers to the target item from the combination of answers to these repeated items. Finally, the assumed birthday probability may be incorrect for a specific sample, for instance when birthday dates are clustered in time or unknown (Sayed et al., 2022). To prevent these issues, it is important to use baseline items that participants perceive as (1) private, (2) neither salient nor weird, and (3) that do not repeatedly involve the same domain. For instance, Sayed et al. (2022) propose using a number sequence randomizer that improves much upon use of birthday items.

### Statistical Model CM2: Between-Participants Information

To use model CM2, the response to the baseline item is asked “directly” in a separate “control group” of survey participants. This control group does *not* respond to the corresponding CM. Thus, ${{\,\mathrm{\mathbb {P}}\,}}(Z_{i} = 1)$ is estimated by simply asking it to a separate group of people, resulting in a two-group (treatment/control) design. Inference relies again on likelihood 2. Model CM2 is rarely applied for the CM (e.g. Jerke et al. 2021). Although it allows a larger variety of non-threatening and non-salient baseline items, it is typically more inefficient than Model M1, because the control group is only used to infer the prevalence of the baseline item. Further, to obtain ${{\,\mathrm{\mathbb {P}}\,}}(U_{i} = 1 | Z_{i} ) = {{\,\mathrm{\mathbb {P}}\,}}(U_{i} = 1 )$ the target and baseline items must be conditionally independent. Although this assumption is common for methodologies such as the List Experiment, it is untestable, and association between the two items may prevent correct estimation of the prevalence of the target item^2^. In contrast, in model CM1 statistical independence is assumed since the responses to the baseline item are conceptually equivalent to the outcome of a randomization device.

### Statistical Challenges of Model 1 and 2

**Fig. 1:**
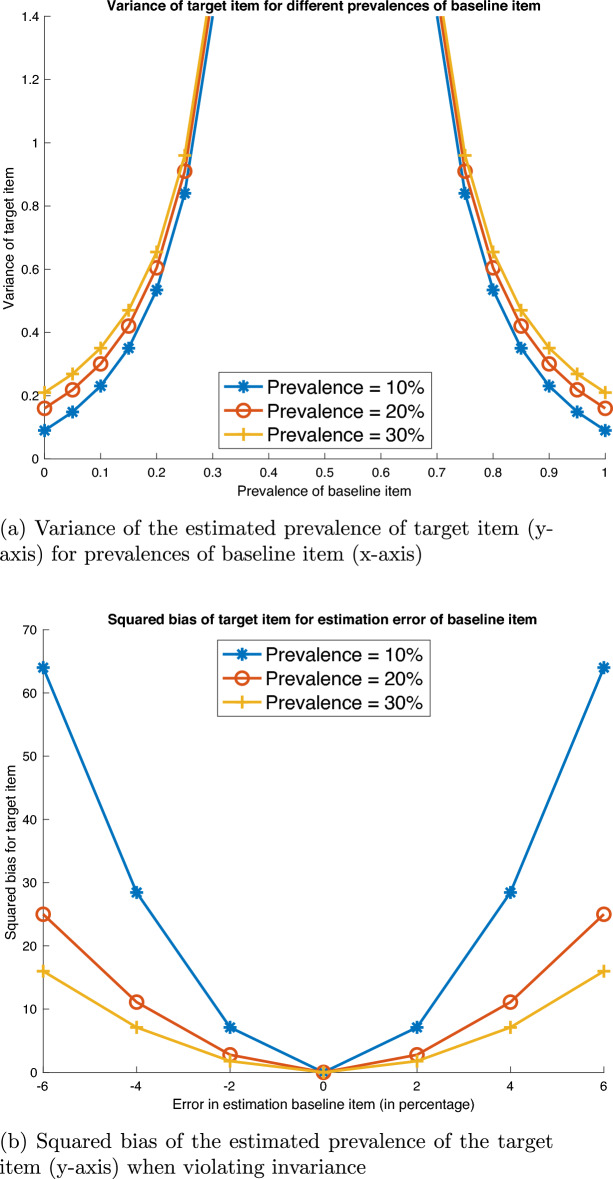
**a** Variance of the estimated prevalence of target item (*y*-axis) for prevalences of baseline item (*x*-axis). **b** Squared bias of the estimated prevalence of the target item (*y*-axis) when violating invariance.

Models CM1 and CM2 have statistical challenges. First, as the prevalence of the baseline item approaches 50 %, the estimated variance of the prevalence $P(U_i=1)$ of the target item increases exponentially. This is illustrated in Fig. 1a, which shows the estimated variance as a function of the prevalence of the baseline item for model CM1 (truncated at 30/70 %); we consider target items of prevalence 10, 20 and 30 %. The estimated variance for model CM2 is even larger, due to measurement error in the baseline item. From a statistical perspective, it is desirable for models CM1 and CM2 to select a baseline item with either rare or very common prevalence (that is, less than 10 % or more than 90 %). However, this would significantly compromise privacy protection and, consequently, compliance with the instructions. For instance, with a baseline item such as “I was born in February”, survey participants might deem the likelihood of this event very low and, as a consequence, refuse to answer truthfully or answer randomly.

Second, the probability of affirming the baseline item $P(Z_i = 1)$, whether assumed (model CM1) or estimated (model CM2), must be correct for the CM. For instance, with model CM2, the probability of affirming the baseline item of ${{\,\mathrm{\mathbb {P}}\,}}(Z_{i} = 1)$ in the separate control group must be the same when administering the CM. This “response invariance” assumption (De Jong and Pieters, 2019) stipulates that the response to the baseline item is not affected by the survey context, such as the presence or absence of other items. This assumption might be violated for a number of reasons. For instance, the assumed prevalence of the baseline item is incorrect (Sayed et al., 2022). Further, survey participants may evaluate the baseline item differently when asked directly vs. when evaluating the paired baseline and target items jointly, due to either more or less attention, or response editing (De Jong and Pieters, 2019; Kuha and Jackson, 2014). A small violation can, however, result in large bias. The squared bias is a popular metrics to assess the quality of an estimator, as one component of the mean-squared error. Figure 1b plots the squared bias when estimating target items with prevalence 10, 20 and 30 % and the “response invariance” assumption is violated. The assumed prevalence of the baseline item is 25 %, which is the median prevalence in published CM studies (see supplemental material H). In the middle of the graph (0 value for *x*-axis), the assumed prevalence (25 %) of the baseline item is correct, hence there is no bias. For nonzero values, the researcher assumes a prevalence of the baseline item of 25 %, but in the sample answering the CM the prevalence is different. For instance, 19 % (− 6 in the *x*-axis of Fig. 1b) or 27 % (+ 2 in the *x*-axis of Fig. 1b). Although these deviation are small in an absolute sense, these can result in large biases: as shown in the figure, the size of the bias is larger if the prevalence of the target item $U_i$ is rare (or very common). Models CM3 and CM4 can reduce the estimated variance of the CM and mitigate violations of the response invariance assumption.

### Statistical Model CM3: Within-Participant Information

Model CM3 uses within-participant information obtained elsewhere in the questionnaire during data collection to predict the response to the baseline item. Assume for now that the response to the baseline item in the CM of a certain participant can be perfectly predicted from other items administered elsewhere in the survey, called “outside-the-CM” items. Then, the response to the target item can be inferred perfectly. For instance, suppose that based on the responses to the outside-the-CM items we can determine with certainty that the answer to the baseline item is “yes”. If the response to the CM is “one yes, one no”, the response to the target item must be “no”, and if the response to the CM is “both yes or both no”, the response to the target item must be “yes”. In practice, perfectly predicting the baseline item (for instance, by administering the same baseline questions) would violate privacy protection of survey participants and be unethical as it would mean lying to them about privacy protection.

In our design, we assume that the outside-the-CM items are correlated with the baseline item, but the correlation is not so strong as to endanger individual privacy protection. Concurrently, it is possible to estimate the average prevalence of the target item $U_i$, while protecting individual privacy. For this model to work, prior knowledge is needed on the relationship between the baseline item and the outside-the-CM items. An option is to use items from a validated multi-item scale with moderate reliability (e.g., Cronbach alpha of .8, as in De Jong and Pieters (2019)). As an example, suppose that the baseline item in the CM is “a. I have a lot of self-discipline”. This item comes from the Conscientiousness facet of a Big Five personality inventory (Goldberg, 1992). Then, other items from the same facet (with five response options: strongly disagree, disagree, neither disagree nor agree, agree, strongly agree) can be used to predict the baseline item (a). Several items from this facet are:

1. I get chores done right away
2. I follow a schedule
3. I leave my belongings around (reverse scored)

The advantage of using within-participant information is that it can potentially reduce the estimated variance of the prevalence of the target item. The within-participant information should allow for more precise estimates, without strictly replicating the baseline item, because that would compromise privacy protection. Additional advantages of this approach are that it obviates the need for a control group and that it can potentially control for invariance and allow for some degree of dependence between the target and baseline items. Further, it allows for a variety of common questions, such as about personality traits, whose true answer is only known to the survey participant, thereby avoiding salient and potentially threatening items. Given that the outside-the-CM items as well as the baseline item are part of a validated scale, an Item Response Theory (IRT) specification is a natural avenue for statistical inference (Fox, 2010).

#### Model CM3a: Item Response Theory Specification

This section provides the formal details of statistical model CM3a. The model accommodates the fact that the response variables are binary (items in the CM) and binary or ordered (items outside-the-CM). We assume that *H* outside-the-CM polytomous items are asked directly elsewhere in the questionnaire. The outside-the-CM items and the baseline item reflect some individual latent trait, denoted as $\theta _{i}$. We employ a specific parametric function to link the trait and the responses, but the model can also be customized for alternative distributions. The observed score to the outside-the-CM items $S_{ih}$ is modeled as: (Samejima, 1969):

5 $$\begin{aligned} {{\,\mathrm{\mathbb {P}}\,}}(S_{ih} = c| \theta _{i}, \alpha _{h}, \varvec{\gamma }_{h}) = \Phi ( \alpha _{h} \theta _{i} - \gamma _{h, c-1}) - \Phi ( \alpha _{h} \theta _{i} - \gamma _{h, c}) \end{aligned}$$

where $\Phi (\cdot )$ denotes the normal cumulative distribution function. The model specifies the conditional probability of a graded response $S_{ih}$ in category $c \in \{1,\cdots , C \}$. The specification in Eq. 5 maps a latent trait parameter $\theta _{i}$ of individual *i* and item-specific parameters $\alpha _{h}$ and $\varvec{\gamma }_{h}$ into an observed pattern of responses. A “trait” here is a general term to indicate some underlying latent construct, such as a personality trait, value, norm or attitude. $\alpha _{h}$ denotes the discrimination parameter and $\varvec{\gamma }_{h}$ denotes the threshold or difficulty parameter (Fox, 2010). The response to the baseline item in the pair is modeled as:

6 $$\begin{aligned} {{\,\mathrm{\mathbb {P}}\,}}(Z_{i} = 1 | \theta _{i}, \alpha _{bas}, \gamma _{bas}) = \Phi (\alpha _{bas} \theta _{i} - \gamma _{bas}) \end{aligned}$$

If no covariates are available to predict the target item and assuming conditional independence of $Z_i$ and $U_i$, then ${{\,\mathrm{\mathbb {P}}\,}}(U_{i} = 1|Z_i) = {{\,\mathrm{\mathbb {P}}\,}}(U_{i} = 1) = \pi$. As earlier, we define $Y_i = 1$ if individual *i* replies that either statement is true when answering the CM and $Y_i = 0$ if individual *i* replies that both or none of the two statements is true. The probability of answering $Y_i = 1$ is thus:

7 $$\begin{aligned} {{\,\mathrm{\mathbb {P}}\,}}(Y_i = 1| \theta _{i}, \alpha _{bas}, \gamma _{bas}, \pi ) = (1-\pi ) \times {{\,\mathrm{\mathbb {P}}\,}}(Z_{i} = 1|\theta _{i}, \alpha _{bas}, \gamma _{bas})+\pi \times {{\,\mathrm{\mathbb {P}}\,}}(Z_{i} = 0|\theta _{i}, \alpha _{bas}, \gamma _{bas}) \end{aligned}$$

Notice that model CM3a does not require the assumption of invariance of the baseline item when estimating the prevalence $\pi$, in contrast to model CM2. This is because with model CM2 the analyst simply plugs in ${{\,\mathrm{\mathbb {P}}\,}}(Z_i = 1)$ in Eq. 1, after having estimated it in a separate control group. In contrast, with model CM3a the analyst jointly estimates the most likely answers to both the target and the baseline items, conditional on the information obtained from the outside-the-CM items. The likelihood for individual responses $Y_i$ and $S_{ih}$ is:

$$\begin{aligned}&\mathcal {L}(Y_i, S_{ih}|\theta _{i}, \alpha _{bas}, \gamma _{bas}, \pi , \alpha _{h}, \varvec{\gamma }_{h},\mu ,\sigma ) \\ {}&\quad = \int \limits _{\Theta } {{\,\mathrm{\mathbb {P}}\,}}(Y_i = 1|\theta _{i}, \alpha _{bas}, \gamma _{bas}, \pi )^{Y_i} (1-{{\,\mathrm{\mathbb {P}}\,}}(Y_i = 1|\theta _{i}, \alpha _{bas}, \gamma _{bas}, \pi ))^{1-Y_i} \\&\quad \left[ \prod _{h=1}^H \prod _{c=1}^C {{\,\mathrm{\mathbb {P}}\,}}(S_{ih} = c| \theta _{i}, \alpha _{h}, \varvec{\gamma }_{h})^{1[S_{ih} = c]} \right] \phi (\theta _i | \mu , \sigma ) d \theta \end{aligned}$$

where $\phi (\cdot )$ is the pdf of a Gaussian distribution. Because we rely on variation in the latent trait $\theta _i$ to identify the threshold parameter $\gamma _{bas}$, stronger identification assumptions are required on the discrimination parameter $\alpha _{bas}$, ensuring that $\alpha _{bas}\gg 0$. As explored in the simulations section, the use of flat or non-informative priors results in a small bias in the estimate of the prevalence $\pi$. This is resolved by eliciting more informative priors, which can be formulated based on existing scale information (Mikkola et al., 2021). Informative priors can be constructed based on external information on the items, such as discrimination parameters of published scales. Alternatively, the items can be administered directly to a separate sample and the resulting IRT estimates can be used to formulate a suitable prior. This is conceptually similar to Model CM4a (presented later), but does not make any assumption on the threshold parameter $\gamma _{bas}$.

The model can be extended to allow for dependence between the target item and the baseline item as follows. Suppose that the analyst is interested in relating the latent trait $\theta _i$, as well as other covariates of interest $\textbf{x}_i$ to the sensitive target item $U_{i}$ (“antecedents”). Using a probit formulation, the probability of answering the target item ${{\,\mathrm{\mathbb {P}}\,}}(U_{i} = 1| \theta _i, \textbf{x}_i)$ affirmatively is:

8 $$\begin{aligned} {{\,\mathrm{\mathbb {P}}\,}}(U_{i} = 1| \theta _i, \textbf{x}_i, \varvec{\beta }) = \Phi (\beta _0 + \beta _\theta \theta _i + \textbf{x}_i^\intercal \varvec{\beta }_X ) \end{aligned}$$

**Fig. 2:**
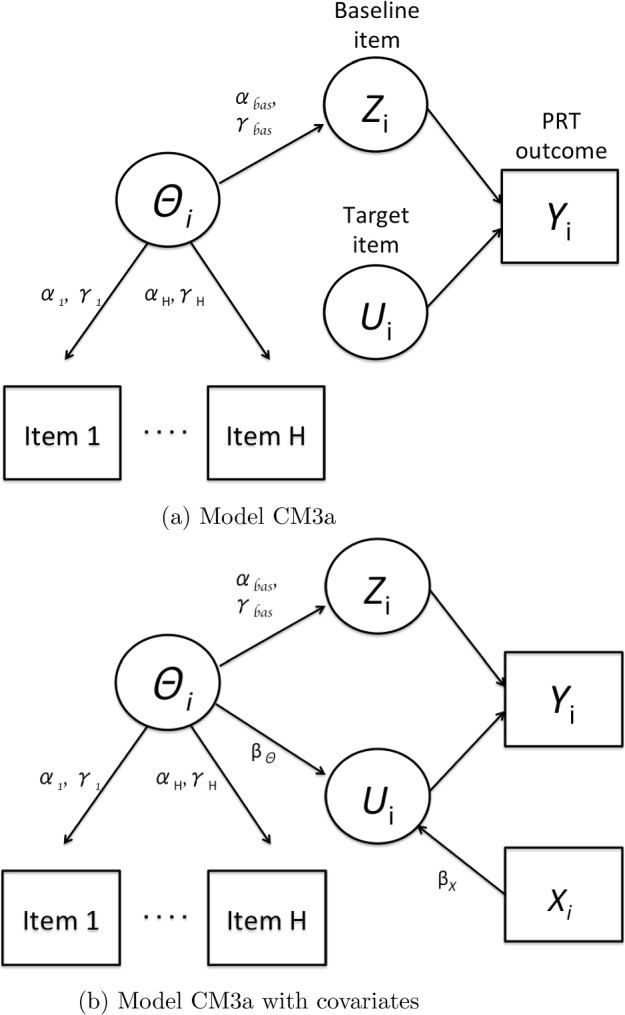
DAGs for model CM3a. *Note* The latent trait $\theta _i$ underlies the responses to the outside-the-CM items 1,..., H and to the baseline item $Z_i$. The baseline item $Z_i$ and the target item $U_i$ jointly determine the CM outcome $Y_i$. In the left model the latent trait $\theta _i$ and other covariates $X_i$ predict the target item $U_i$. DAGs for model CM3a.

Then, ${{\,\mathrm{\mathbb {P}}\,}}(U_{i} = 1 | Z_{i} )$ from Eq. 1 can be estimated, since the answer to $Z_i$ depends on $\theta _i$ (De Jong and Pieters, 2019). We examine later using simulations to what extent the model parameter $\beta _{\theta }$ can be correctly estimated. Figure 2a presents model CM3a in a directed acyclic graph when assuming conditional independence, and Fig. 2b presents model CM3a in a directed acyclic graph when relating some antecedents covariates $\textbf{x}_{i}$ and the latent trait $\theta _i$ to the sensitive target response. Extensions and model modifications are possible. Whereas we use a Gaussian link for Eqs. 5, 6 and 8, different response functions may be used, such as logistic or partial credit models. Further, one could alternatively predict the baseline item based on other covariates, which is examined in the next section.

#### Model CM3b: Binary Response Model

Model CM3a assumes that baseline and outside-the-CM items are selected from a validated scale. However, the baseline item might not necessarily come from such a scale, yet may be predicted on the basis of some observed set of covariates $\textbf{x}_i$ (Kuha and Jackson, 2014). Given a link function $g(\cdot )$, such as $g(\cdot ) = \Phi (\cdot )$:

9 $$\begin{aligned} {{\,\mathrm{\mathbb {P}}\,}}(Z_{i} = 1 | \textbf{x}_i, \varvec{\delta }) = g(\delta _0 + \textbf{x}_i^\intercal \varvec{\delta }_{bas}) \end{aligned}$$

As with Model CM3a, the baseline item is administered only within the CM, thus by using an indirect question. The probability of answering $Y_i = 1$ is:

10 $$\begin{aligned} {{\,\mathrm{\mathbb {P}}\,}}(Y_i = 1| \pi , \textbf{x}_i, \varvec{\delta }) = (1-\pi ) \times {{\,\mathrm{\mathbb {P}}\,}}(Z_{i} = 1 | \textbf{x}_i, \varvec{\delta }) + \pi \times {{\,\mathrm{\mathbb {P}}\,}}(Z_{i} = 0 | \textbf{x}_i, \varvec{\delta }) \end{aligned}$$

The model can be easily extended to predict the target item the using available covariates. As with Model CM3a, alternative parametric functions may be used. In the simulation section, we discuss to what extent Models CM3a and CM3b can correctly estimate the prevalence of the target item.

### Statistical Model M4: Hybrids

Hybrid models can be implemented combining models CM2 and CM3, that is, using both information from a separate control group (between-participants) and within the individual (within-participants). When applying model CM4, a separate control group answers directly the baseline item. The between-participants information can then be leveraged for inference, obtaining more precise estimates of the baseline item parameters. The price of additional precision is however having to invoke the assumption of response invariance of the baseline item, as in model CM2. We propose two statistical models, namely models CM4a and CM4b:

- *Model CM4a (IRT model)*: If, as assumed in model CM3a, the baseline and the outside-the-CM items all come from a validated multi-item scale, an IRT model can be estimated based on Eqs. 5 and 6. The statistical framework is thus equivalent to model CM3a. We, however, emphasize that with model CM3a no external information is used for the threshold parameter $\gamma _{bas}$ (in contrast to model CM4a)
- *Model CM4b (binary response model)*: The statistical framework of model CM4b is equivalent to model CM3b.

#### p-Groups Design to Minimize Variance

If the response invariance assumption is not a concern, a p-groups design is the most efficient way to minimize the estimated variance of the target item in the CM. The use of p-groups designs has been proposed for List Experiments (Glynn, 2013; Blair and Imai, 2012). In a p-group design, the analyst splits the sample in p groups of equal size. Each group receives the same target item but a different baseline item. Each baseline item is then asked directly in the other groups. If based on model CM4a, the baseline and the outside-the-CM items should all come from the *same* validated scale. If the analyst does not wish to use a baseline item from a validated scale, model CM4b can be used for analysis, relating the baseline item to other observed variables. The p-group design improves efficiency over all other models and also allows detecting potential violations of model assumptions. This is explored more in detail in the next four MC simulations, examining the performance of the various models.

## Monte Carlo Simulations of CM Performance

Four MC simulations are conducted. The first MC simulation shows that “known prevalence” items used with the popular model CM1 have lower efficiency (higher estimated variance) than a p-groups design (with *p* = 2) with models CM4a and CM4b. The second MC simulation examines the capacity of models CM3a and CM3b to correctly estimate the prevalence of the target item. These models have the advantage that they do not strictly rely on external information on the prevalence of the baseline item, such as a sample average from a separate group or an assumed probability. This relaxes the response invariance assumption, as discussed in the third MC simulation. Finally, the fourth MC simulation examines the possibility to test and account for dependence between the baseline and target item with the IRT structure of models CM3a and CM4a.

### Comparison of Models CM1, CM4a and CM4b: Efficiency

In this MC simulation, the sample size is fixed to $n_\textrm{CM} = 1000$^3^. The experimental design is 3 (prevalence $\pi$) by 3 (reliability for CM4a; pseudo-$R^2$ for CM4b) by 5 (threshold $\gamma _{bas}$ for CM4a; intercept $\delta _0$ of baseline item for CM4b), for a total of 45 cells, with 200 simulations for each cell. The first factor, the prevalence of the target item $U_i$, is set at $\pi \in \{.1,.2,.3\}$ (results are symmetric for $\pi \in \{.9,.8,.7\}$). Baseline item parameters are varied for models CM4a ($\alpha _{bas}$, $\gamma _{bas}$), using Eqs. 5 to 7, and CM4b ($\delta _{bas}$, $\delta _0$), using Eq. 9. We use a 2-groups design where one item has threshold $\gamma _{bas}$ (intercept $\delta _0) \in \{1.5, 1.25, 1,.75,.5\}$, and the other item has symmetric threshold $\gamma _{bas}$ (intercept $\delta _0) \in \{-1.5, -1.25, -1, -.75, -.5\}$. For model CM4a, we draw discrimination parameters $\alpha _{h}$ and $\alpha _{bas}$ corresponding to scale reliability values of $\{.6,.7,.8\}$. Similarly, for model CM4b we use a probit specification with intercept where we vary the coefficient $\delta _{bas}$ of a single explanatory variable $x_i$ to obtain a pseudo-$R^2$ of $\{.1,.2,.3\}$.

To ensure a fair comparison, the corresponding prevalence of the baseline item is used to simulate data with model CM1. To illustrate this, Table 3 gives the prevalence of the baseline item for some combinations of baseline item parameters. Cases such as $\gamma _{bas}<-1.5$ ($\delta _0 <-1.5$) are not examined because these values imply that the prevalence of the baseline item is more than 90 %, which is problematic. If survey participants feel that the endorsement of the baseline item is so common or rare—and thus not privacy protecting—they are more likely *not* to comply with the instructions. In practice, the median prevalence for baseline items in empirical applications is approximately 25 (75) % (see supplemental material H), corresponding to $\gamma _{bas}$ ($\delta _0) \approx \pm 1$.

**Table 3:** Aggregate probability of baseline item: varying combinations of intercept $\gamma _{bas} (\delta _0$) and of coefficient $\alpha _{bas} (\delta _{bas})$.

$\alpha _{bas} (\delta _{bas})$	$\gamma _{bas} (\delta _0$)									
	− 2	− 1.5	− 1.25	− 1	− 0.5	0.5	1	1.25	1.5	2
0.5	96.3	91.3	87.0	81.2	67.3	32.7	18.8	13.0	8.7	3.7
0.75	94.6	88.8	84.2	78.7	65.7	34.3	21.3	15.8	11.2	5.4
1	92.5	85.6	81.4	75.7	63.6	36.4	24.3	18.6	14.4	7.5
1.25	89.3	82.9	78.1	73.6	62.3	37.7	26.4	21.9	17.1	10.7

**Table 4:** Simulation study: percentage decrease in estimated variance when using model CM4a (left) and model CM4b (right) versus model CM1.

	Model CM4a							Model CM4b				
Scale reliability							pseudo-$R^2$					
$\pi =$ 10 %	$\pm 1.5$	$\pm 1.25$	$\pm 1$	$\pm 0.75$	$\pm 0.5$		$\pi =$ 10 %	$\pm 1.5$	$\pm 1.25$	$\pm 1$	$\pm 0.75$	$\pm 0.5$
0.1	− 1.7	− 8.3	− 17.3	− 32.7	− 56.8		0.1	15.1	16.2	14.4	6.8	− 19.9
0.2	− 23.9	− 33.1	− 45.3	− 60.4	− 77.4		0.2	1.2	− 2.8	− 10.5	− 21.9	− 47.4
0.3	− 50.4	− 58.9	− 70.4	− 80.6	− 90.1		0.3	− 13	− 21	− 30.8	− 45.4	− 65.9
Avg.	− 25.3	− 33.4	− 44.4	− 57.9	− 74.8		Avg.	1.1	− 2.6	− 8.9	− 20.2	− 44.4
$\pi =$ 20 %							$\pi =$ 20 %					
0.1	− 1.3	− 6.7	− 15.6	− 31.8	− 55.9		0.1	7.1	7.8	6.6	1.9	− 15.3
0.2	− 16.5	− 26	− 38.8	− 56.5	− 75.3		0.2	0.7	− 2.6	− 9.3	− 21.4	− 43.6
0.3	− 38.5	− 49.5	− 62.5	− 76.5	− 88.2		0.3	− 7.6	− 14.2	− 24.7	− 40	− 62.2
Avg.	− 18.8	− 27.4	− 38.9	− 55	− 73.2		Avg.	0.1	− 3	− 9.1	− 19.8	− 40.4
$\pi =$ 30 %							$\pi =$ 30 %					
0.1	− 3.4	− 8.5	− 17.4	− 33.6	− 57.5		0.1	2.2	2.2	− 0.3	− 5.4	− 21.7
0.2	− 14.9	− 23.8	− 37	− 55.2	− 75.6		0.2	− 1.5	− 4.3	− 11.4	− 23	− 46
0.3	− 33.2	− 46.2	− 59.7	− 74.4	− 87.7		0.3	− 7.1	− 12.9	− 23.1	− 39.2	− 62.1
Avg.	− 17.2	− 26.2	− 38	− 54.4	− 73.6		Avg.	− 2.1	− 5	− 11.6	− 22.5	− 43.2

Sample size is *N* = 1000. We vary the coefficient reliability of the scale (rel.) or the pseudo-$R^2$ across rows and the intercept $\gamma /\delta _0$ across columns. 200 simulations are run for each cell.

Table 4 shows the percentage change in average estimated variance of the prevalence of the target item when applying a 2-groups design with model CM4a (left) or model CM4b (right) vs. model CM1. A negative value implies that the variance of the target item is higher when estimated with model CM1, as compared to model CM4a or CM4b. Thus, Table 4 shows that model CM4a and CM4b are almost always more efficient than model CM1, reducing the posterior variance of the target item by as much as 90 %. Model CM1 may have better performance only if the reliability is low (less than.6) or the pseudo-$R^2$ is low (less than.2).

The exact gains depend on the specific combination of parameters. In particular, if the reliability of the scale for model CM4a or the pseudo-$R^2$ for model CM4b is higher, the correlation between the baseline item and the outside-the-CM items is larger. In this scenario, then, model CM1 performs much worse than models CM4a and CM4b. This is because the baseline item is more sensitive to variability in the latent trait $\theta _i$ or the covariate $x_i$, and hence, the within-participant information is more effective for estimation. Second, if the threshold parameter $\gamma _{bas}$ (intercept $\delta _0$) is near 0, the probability of affirming the baseline item approaches 50%, and the relative performance of model CM1 is worse.

The median prevalence of baseline items in empirical applications is approximately 25 (75) %, which corresponds to the choice $\gamma _{bas}$ ($\delta _0$) $= \pm 1$. Depending on other model parameters, the reduction in estimated variance is approximately 40 % for model CM4a and 10 % for model CM4b. This large decrease in variance justifies the somewhat more complex modeling of CM4a and CM4b. The added Python and MATLAB code and user app mitigate implementation issues by providing straightforward implementation of models CM4a and CM4b.

#### Analysis of Models CM3a and CM3b

In this section, we show that models CM3a and CM3b correctly recover the target prevalence, although their performance hinges on how accurately the outcomes of the baseline item are predicted. The experimental design is 3 (prevalence $\pi$) by 3 (model CM3a with either non-informative or informative priors, CM3b) by 3 (reliability / pseudo-$R^2$). We vary the reliability of the scale for model CM3a as $\{.6,.7,.8\}$ and the pseudo $R^2$ for model CM3b as $\{.1,.2,.3\}$. The baseline threshold parameter $\gamma _{bas}$/intercept $\delta _0$ is fixed to 1.

**Fig. 3:**
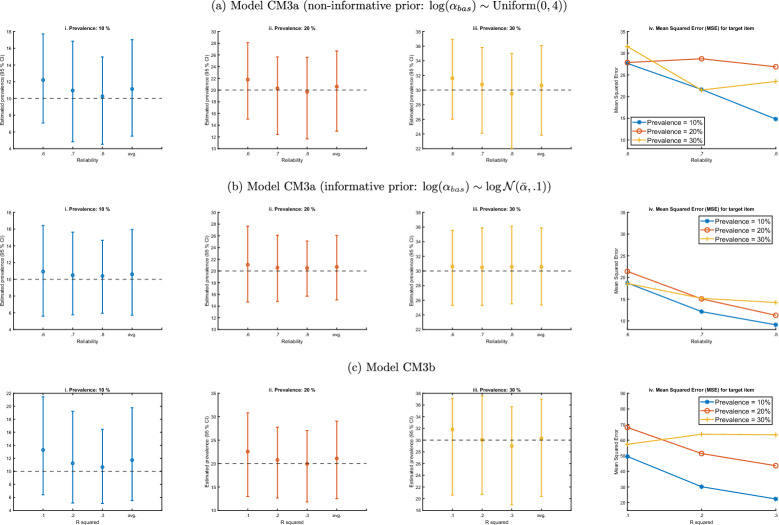
Estimation of prevalence of target item with models CM3a and CM3b.

We examine two separate implementations of model CM3a: one with non-informative prior for the discrimination parameter ($\alpha _{bas} \sim \text {Uniform}(0, 4)$, Fig. 3a) and one with informative priors, based on the scale reliability (Fig. 3b)^4^. Informative priors for the baseline discrimination parameters can be assumed since the items are selected from validated scales with known reliability. Plausible ranges for the discrimination parameters are often available as well in published studies. Results for Model CM3b are shown in Fig. 3c. The figures on the left show the 95 % confidence interval of estimated prevalences across simulations, assuming a true prevalence of 10 % (subfigures i), 20 % (subfigures ii) and 30 % (subfigures iii). Thus, all models seem generally suitable to correctly estimate the assumed prevalence if the reliability is above .6–.7 or the pseudo-$R^2$ is above .2–.3. There seems to be a small bias with model CM3a when the reliability is low; this is mitigated by using more informative priors on the discrimination parameter $\alpha _{bas}$. We emphasize that we do not use an informative prior for the location parameter $\gamma _{bas}$, and hence, the model makes weaker assumptions than model CM4a. Subfigures iv show a decrease of the mean-squared error if the reliability or the pseudo-$R^2$ increases.

#### Comparison of Models CM3a and CM4a: Violations of Response Invariance and Bias

**Fig. 4:**
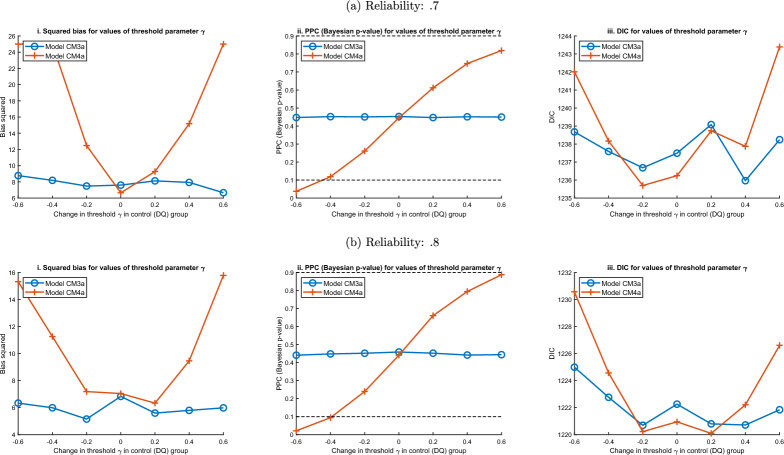
Average bias squared, PPC and DIC when violating invariance with models CM3a and CM4a.

The previous simulations assume that survey participants evaluate the baseline item similarly, regardless of whether it is administered directly or in a CM (that is, jointly with the target item). In the third MC simulation, the bias is examined when this assumption of response invariance is violated. We focus on the IRT models CM3a and CM4a. The following model parameters are used: $\gamma _{bas} = -1$, reliability.7 (Fig.  4a) and.8 (Fig.  4b). In the *x*-axis of the figures, $\gamma _{bas}$ gradually changes *only* in the separate control (DQ) group by increments of .2. A larger change (e.g., $\pm .6$) corresponds to a more severe violation of response invariance.

In subfigures i, the estimation bias in model CM4a increases as the deviation from response invariance worsens. This is because the prevalence estimate of the baseline item in the control group differs from its estimate in the CM. In contrast, model CM3a correctly estimates the prevalence of the target item. This is because it does not rely on estimates from a separate control group, but only on within-participant information.

#### Predictive Checks and Information Criteria to Detect Invariance

Violating the assumption of response invariance will worsen performance of models CM4 (as well as CM2). Model CM3a is robust to invariance. However, model CM4a is typically more efficient than model CM3a. How can we check whether model assumptions are satisfied and select an appropriate model? With models CM1 and CM2, the response invariance assumption is untestable. However, *p*-values of posterior predictive checks (PPC) can be used to alert to violations of invariance *with model CM4a*. These *p*-values indicate whether the model predictions can replicate the observed percentage of “same/different” answers to the CM (Gelman et al., 1996). The reported *p*-values should not be extremely different from.5 when applying a model (e.g., less than.1 or more than.9). Subfigures ii (in Fig. 4) show the average PPC for model CM4a given changes of the parameter $\gamma _{bas}$ in the DQ group. When response invariance is violated more severely (e.g., for a value of the *x*-axis close to $\pm .6$) the PPC for model CM4a approaches 0 or 1. PPC can also be used to detect violations of the model assumptions beyond response invariance, as explored later.

Although model CM3a is more robust to the response invariance assumption, it is typically less efficient than model CM4a, as it does not leverage information from a separate DQ group. The deviance information criterion (DIC) can be used for model selection (Spiegelhalter et al., 2002). Subfigures iii show the DIC for models CM3a and CM4a. If the violation of response invariance is mild, the DIC favors using model CM4a, due to its higher efficiency. However, if the squared bias increases, the DIC favors using model CM3a, as it is robust to violations of response invariance.

#### Conditional Independence of Target and Baseline Items

**Fig. 5:**
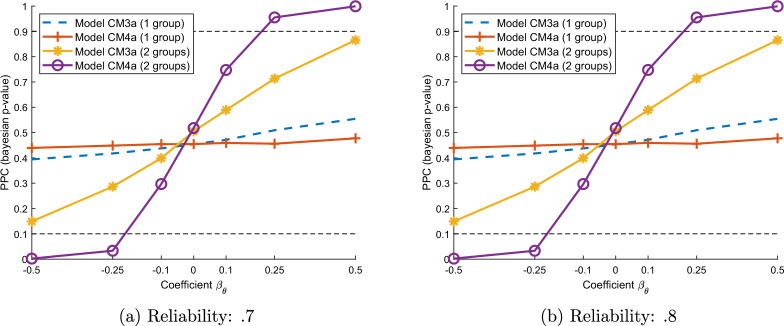
Bayesian posterior predictive check (PPC) when violating conditional independence (varying value of coefficient $\beta _{\theta }$ in *x*-axis).

Conditional independence of the target and baseline items simplifies identification of the prevalence of the target item. This section explores to what extent it is possible to test and account for dependence using IRT modeling, assuming that the baseline and target item are related as specified in Eq. 8. Violations of this “conditional independence” assumption can also be detected with PPC checks for model CM3a and CM4a (without modeling the relationship between the latent trait $\theta _i$ and the latent target item $U_i$). We vary the coefficient $\beta _{\theta }$ in Eq. 8 between − .5 and .5, leading to more or less dependence between the target and baseline items. $\beta _{\theta }=0$ implies statistical independence. Figure 5 shows the average PPC criterion across simulations for models CM3a and CM4a, using both single group (*p* = 1) and 2-groups design (*p* = 2). Hence, model CM4a is most sensitive to violations of the conditional independence assumptions when using 2 groups: for instance, the average PPC is more (less) than .95 (.05) when the coefficient $\beta _{\theta }$ is larger (smaller) than.25 (− .25). The PPC of Model CM3a with a 2-group design is also sensitive to violations of conditional independence, but less so than model CM4a.

**Fig. 6:**
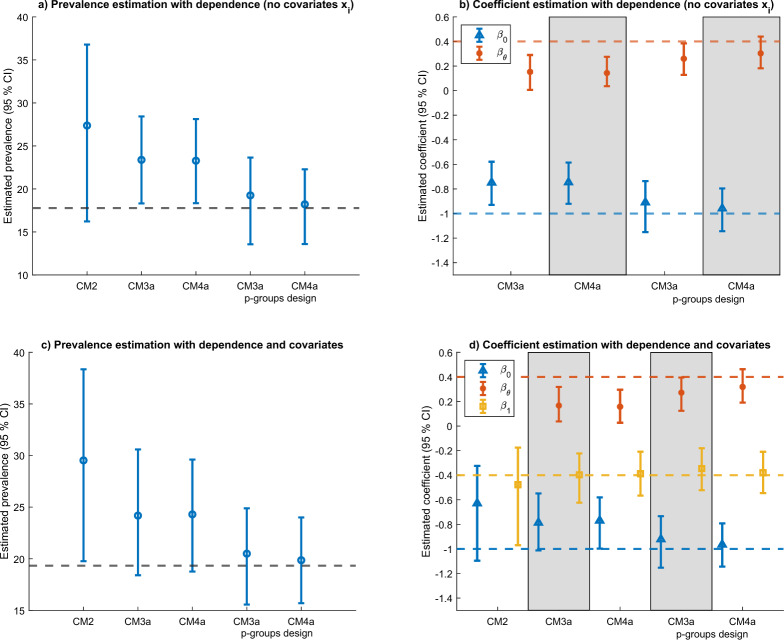
Estimation of statistical dependence with reliability. 7.

**Fig. 7:**
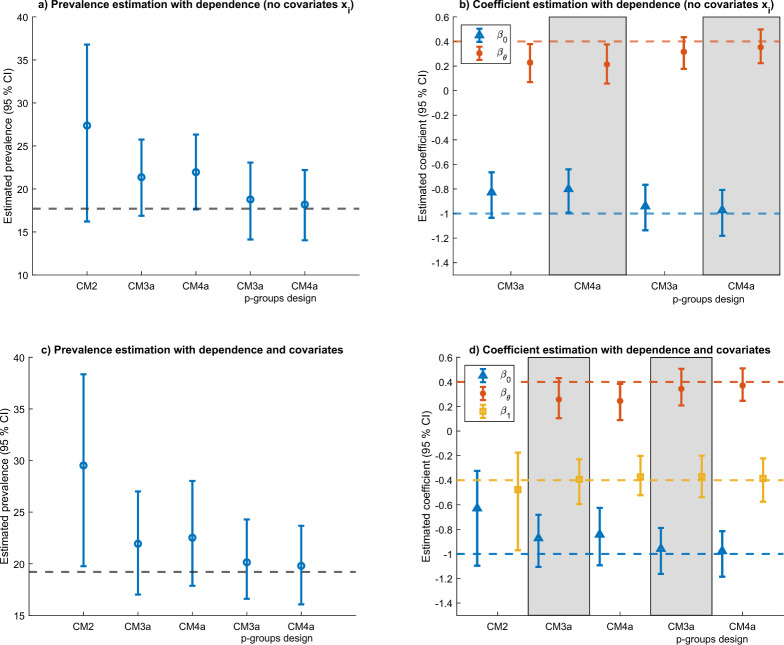
Estimation of statistical dependence with reliability .8.

**Fig. 8:**
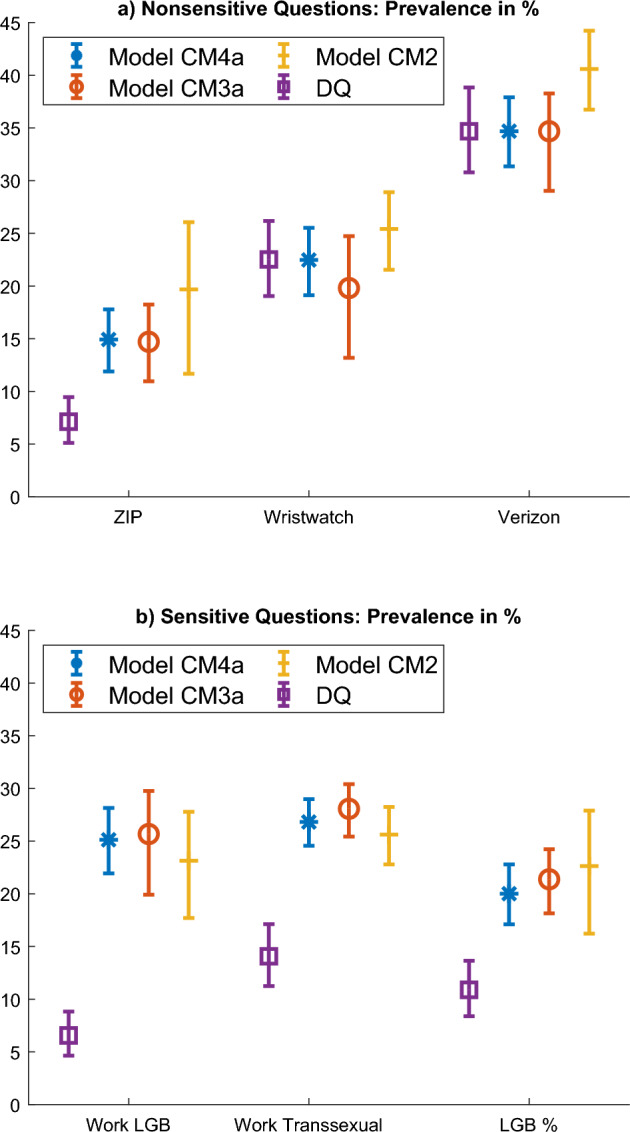
Error bar plots illustrate DQ and models CM4a, CM3a and CM2. *Note* Average prevalence for (top) non-sensitive target items and (bottom) sensitive target items. Error bar denotes 95% credibility interval.

We next study whether each model is suitable to estimate dependency between the target and baseline item, assuming that the probit model of Eq. 8 is correctly specified. We assume a probit link with parameters: $\beta _0 = -1$, $\beta _{\theta } =.4$. We then also include a single regressor $x_i \sim \mathcal {N}(0,1)$ with parameter $\beta _{1} = -.4$ (subfigures c and d). We test models CM2, CM3a and CM4a in a single group design (*p* = 1), and models CM3a and CM4a with a 2-groups design (*p* = 2). We use discrimination parameters corresponding to reliability of.7 (shown in Fig. 6) and.8 (shown in Fig. 7).

Subfigures a and c show to what extent we can recover the correct population prevalence of the target item. The subfigures reveal that when the two items (baseline and target) are correlated, model CM2 is unsuitable to recover the correct target item prevalence. In this scenario, the bias is upward because $\beta _{\theta } =.4 > 0$. The bias becomes smaller when using models CM3a and CM4a, but it is negligible only when implemented in a 2-groups design. Subfigures b and d examine estimation of the coefficients in the probit regression model. The regression is not estimated for model CM2 in subfigure b since there is no regressor. When estimating models CM3a and CM4a there is a downward bias for the coefficient $\beta _{\theta }$, which is reduced when using scales with higher reliability (e.g., see subfigure 5d). This suggests that the coefficient $\beta _{\theta }$ can also be used to test for (linear) dependence between the target and the baseline item under IRT modeling. However, some care must be taken with this test since a) it requires collecting data with a p-groups design and b) the simulations suggests that it may be biased toward zero, if using scales with lower reliability.

The simulation experiments provide the following insights. First, models CM4a and b improve efficiency over the more commonly used model CM1 and consequently CM2. Second, models CM3a and b can correctly estimate the prevalence of the target item, without relying on external information about the prevalence of the baseline item via a control group. However, their performance crucially depends on the reliability of the scale used or accurate prediction of the baseline item: we recommend using scales of reliability .7 – .8. Third, only model CM3 is robust to violations of response invariance. Fourth, and finally, IRT models CM3a and CM4a can detect and model potential association between the baseline and target item, although we have to acknowledge that their performance also depends on correct parametric specification.

## Empirical Application: Workplace Attitudes Toward the LGBT Community

Models CM2, CM3a and CM4a are used to examine sensitive workplace attitudes toward the LGBT community, and the size of the LGBT population. Prior research (Schnell and Thomas, 2021; Sagoe et al., 2021) has already examined the basic model CM1 in detail; hence, we improve and complement the existing literature by showing how to use models CM2, CM3a and CM4a. All data and codes are publicly available at the OSF repository and can be accessed at https://osf.io/wprtf/?view_only=6917442acc3047f1aee700deb24fe45e. The study was approved by the appropriate IRB but not preregistered.

Data were collected in June 2019 with an online survey using a convenience sample of 3258 US participants aged above 18 on Amazon MTurk (https://www.mturk.com/). The survey completion rate was approximately 92.7 %. The sample is 50% female, the median age is between 26 and 35 years old. 58.8% had an education at the level of a bachelor degree or higher. Survey participants were randomly assigned to one of two conditions: direct question (DQ; *n* = 531, 16.3 %) or CM (*n* = 2727; 83.7 %). Survey participants in the DQ condition were directly asked both the target and the baseline items, thus functioning as control group. The following sensitive items were administered:

1. I would have a problem working with an openly lesbian, gay, or bisexual coworker
2. I would have a problem working with a transsexual coworker
3. I am an LGBT individual

The IRT-based models CM3a and CM4a are compared with model CM2 using “face validity” criteria: a “placebo check” with non-sensitive questions and a “more is better” criterion with sensitive questions^5^, similar to Coffman et al. (2016) and Höglinger and Diekmann (2017). Survey participants should answer truthfully under the CM and misreport under direct question when the target item is sensitive. Further, survey participants should respond similarly to the CM and direct question techniques when the target item is not sensitive. This is tested by administering three items presumed to be non-sensitive before the sensitive items (namely, “The ZIP code of my home address begins with 6”, “I am wearing a wristwatch” and “I am a Verizon client”, based on Coffman et al. (2016)). The paired baseline items are personality traits which should be unrelated to the target items of interest (see supplemental material E) and which are shown in Table 6. Each model is therefore evaluated on the basis of three criteria:

- *Placebo check*. Which model has the smallest differences in prevalence of the target item between CM and DQ for the non-sensitive items.
- *More is better*. Whether the model has significantly larger prevalence for the target item under CM as compared to DQ.
- *Estimated variance*. Which model has lower estimated variance for the prevalence of the target item.

This approach lacks response validation at the individual level and has been thus criticized in the literature (Höglinger and Jann, 2018; Walzenbach and Hinz, 2019): care must be taken with interpreting the results since we do not have evidence on the actual individual responses. The plausibility of the invariance assumption is investigated using the PPC and model selection is implemented using the DIC. Results are obtained using 25000 draws for burnin and 25000 draws for computing posterior statistics using the MATLAB codes.

### Results

Table 5 presents the estimated percentage of “yes” for non-sensitive (items a, b and c) and sensitive items (items d, e and f) using the different models. The first column in the table presents the results with model CM2 and then the difference with reported behavior under direct question. The next columns present the results for models CM3a and CM4a. Figure 8 illustrates this with a bar plot.

**Table 5:** Attitudes toward LGBT people: estimates from models CM2, CM3a and CM4a.

Target items	Model CM2		Model CM3a		Model CM4a		
	1. CM	2. CM-DQ	3. CM	4. CM-DQ	5. CM	6. CM-DQ	7. DQ
Non-sensitive target items							
a. The ZIP code of my home	19.7	12.5***	14.7	7.6***	14.9	7.8***	7.1
address begins with 6	(3.7)	(3.9)	(1.9)	(2.2)	(1.5)	(1.9)	(1.1)
b. I am wearing a	25.4	2.9	19.8	− 2.7	22.5	0.1	22.5
wristwatch right now	(1.9)	(2.6)	(3.0)	(3.5)	(1.6)	(2.4)	(1.8)
c. I am a Verizon customer	40.6	5.9*	34.6	− 0.1	34.7	0	34.7
	(1.9)	(2.8)	(2.3)	(3.1)	(1.6)	(2.6)	(2.1)
Sensitive target items							
e. I would have a problem working	23.1	16.6***	25.7	19.1***	25.1	18.6***	6.6
with a colleague who is LGB	(2.6)	(2.8)	(2.5)	(2.7)	(1.6)	(1.9)	(1.1)
d. I would have a problem working	25.6	11.6***	28.1	14.0***	26.8	12.7***	14.1
with a colleague who is transsexual	(1.4)	(2)	(1.3)	(2)	(1.1)	(1.9)	(1.5)
e. I am an LGBT individual	22.6	11.7***	21.4	10.5***	20	9.1***	10.9
	(3)	(3.3)	(1.5)	(2.0)	(1.4)	(2)	(1.3)
Sample size	*N* = 3258		*N* = 2727		*N* = 3258		*N* = 531

Each column reports the average prevalence in % of the target item and the difference with direct question (DQ). *: 95% credible interval (CI) does not include zero; **: 97.5% CI does not include zero; ***: 99% CI does not include zero.

**Table 6:** IRT estimates for discrimination parameter, threshold parameter, and probability of affirming the baseline item.

Baseline Items	CM (Model CM3a)			DQ			CM (Model CM3a)—DQ		
	1. Disc.	2. Diff.	3. Prob.	4. Disc.	5. Diff.	6. Prob.	7. Disc.	8. Diff.	9. Prob.
a. I keep in	1.11	− 0.68	67.2	1.22	− 0.87	71	− 0.11	0.2	− 3.8
the background	(0.13)	(0.08)	(1.3)	(0.14)	(0.1)	(1.6)	(0.19)	(0.13)	(2)
b. I make people	0.86	− 1.02	77.7	0.72	− 1.14	82.4	0.14	0.13	− 4.7
feel at ease	(0.15)	(0.16)	(2.3)	(0.1)	(0.09)	(1.5)	(0.18)	(0.18)	(2.7)
c. I am relaxed	1.24	− 0.69	66.3	0.86	− 0.96	76.7	0.38	0.27	− 10.4***
most of the time	(0.31)	(0.2)	(2.9)	(0.11)	(0.09)	(1.6)	(0.33)	(0.22)	(3.4)
e. I have a lot of	0.94	− 1.13	79.2	0.85	− 0.95	76.4	0.09	− 0.18	2.7
self-discipline	(0.18)	(0.19)	(2.5)	(0.1)	(0.09)	(1.6)	(0.21)	(0.22)	(3)
d. I like order	0.96	− 2.4	95.5	0.74	− 1.55	89.5	0.23	− 0.85	6**
	(0.21)	(0.39)	(1.7)	(0.11)	(0.11)	(1.2)	(0.24)	(0.4)	(2.1)
f. I am quiet	1.21	− 0.89	71.3	1.15	− 0.98	73.9	0.06	0.08	− 2.6
around strangers	(0.17)	(0.12)	(1.6)	(0.12)	(0.1)	(1.6)	(0.21)	(0.15)	(2.2)
Sample size	*N* = 2727			*N* = 531			*N* = 3258		

#### Placebo Check: Nonsensitive Items

We begin by examining the performance of the CM with non-sensitive target items. Model performance is better the closer it is to the prevalence of the target items estimated in the control group. All 95 % credibility intervals of the differences discussed in this section exclude 0, unless specified otherwise. Model CM2 (Table 5) consistently results in overreporting as compared to DQ. In particular, for the first presumed non-sensitive item (“The ZIP code of my home address begins with 6”) 19.7 % of survey participants said “yes” with the CM, while only 7.1 % did so with DQ. Furthermore, the third presumed non-sensitive item (“I am a Verizon client”) produced overreporting, where 40.6 % of survey participants indicated “yes” with the CM, against only 34.7 % with DQ. Similar false-positive problems using model CM2 affect applications of the CM also when using model CM1 (Höglinger and Diekmann, 2017; Höglinger and Jann, 2018).

Model CM3a fares better. Only *item a.* (about the ZIP code) results in a difference with DQ: 14.7 % of survey participants affirm the item with the CM, whereas only 7.1 % do so with DQ. However, the difference is much smaller as compared with model CM2 (namely, 7.6 % vs. 12.5 %), although its 95 % credibility interval still excludes 0. The results of model CM4a are a weighted average of model CM2 and CM3a. Only *item a.* once again results in a clear difference with DQ, as 14.9 % of participants affirm the item with the CM. The estimated prevalence for the other two items is almost identical to the DQ result. This provides some aggregate-level evidence on the effectiveness of models CM3a and CM4a to predict the baseline item.

#### More is Better: Sensitive Items

Responses to sensitive questions differ much between DQ and the CM. For instance, with direct question, 6.6 % of the survey participants report having a problem working with an openly LGB colleague. The percentage estimated with model CM2 is a much higher 23.1 %, providing evidence of underestimation when asking this item directly. With direct question, 14.1 % of the survey participants report having a problem working with a transsexual colleague. The percentage estimated with model CM2 is much higher, with a mean posterior probability of 25.6 %. Reporting on one’s own sexuality also differs widely between questioning techniques. In the DQ group, 10.9 % of survey participants self-identify as being a LGBT person. However, using model CM2, an estimated 22.6 % of this sample self-identified as LGBT.

Similarly, when looking at model CM3a, 25.7 % of the participants report having a problem working with an openly LGB colleague, 28.1 % of the sample report having a problem working with a transsexual colleague, and 21.4 % of the sample self-identify as being a LGBT person. Model CM4a yields similar results.

#### Efficiency of the CM

Table 7 reports the percentage change in estimated variance for model CM3a and CM4a vs. Model CM2. In line with the Monte Carlo simulations, the IRT-based models generally achieve higher efficiency. Model CM4a, in particular, decreases the estimated variance from 24.9 to 83.6 % as compared to model CM2. The gains are noticeably more marked as the prevalence of the baseline item approaches 50 % and as the discrimination parameter $\alpha _{bas}$ is larger (for instance, see items a and f in Table 6).

**Table 7:** Posterior estimated variance of prevalence of target item for models CM2, CM3a and CM4a, with corresponding % change (model CM2 as reference).

	Model CM2	Model CM3a (% change)		Model CM4a (% change)	
a. The ZIP code of my home	13.8	3.5	− 74.7	2.3	− 83.6
address begins with 6					
b. I am wearing a	3.5	9.2	160.0	2.6	− 24.9
wristwatch right now					
c. I am a Verizon customer	3.7	5.4	47.4	2.7	− 25.2
e. I would have a problem working	6.7	6.3	− 5.8	2.5	− 62.1
with a colleague who is LGB					
d. I would have a problem working	1.9	1.6	− 18.0	1.3	− 34.5
with a colleague who is transsexual					
e. I am an LGBT individual	9	2.4	− 73.5	2.1	− 76.7

#### Assumption of Invariance and Information Criteria

Table 8 provides PPC and DIC values of the models for the full sample. The PPC should not diverge significantly from.5 when using model CM4a: the results suggest that the assumption of invariance is inappropriate when the target item is “c. I am a Verizon client”. The PPC for Model CM4a is approximately 1, implying that the model predictions do not match the observed probability of affirming that “the responses to the two statements are different”. This might explain why applying Model CM2 with this item results in significant over-reporting (see Table 5). Furthermore, the DIC indicates that models CM3a and CM4a should be preferred over model CM2. When invariance is violated, as with item c, the DIC indicates selecting model CM3a (for instance, with item c). In conclusion, there is some evidence that the assumption of invariance may be violated, leading to inappropriate implementation of models CM2 and CM4a for these items.

**Table 8:** Model selection for full sample: PPC and DIC.

	Model CM2		Model CM3a		Model CM4a	
	1. PPC	2. DIC	3. PPC	4. DIC	5. PPC	6. DIC
Non-sensitive target items						
a. The ZIP code of my home	0.5	3607.5	0.39	3184.2	0.34	**3184.0**
address begins with 6						
b. I am wearing a	0.5	3499.3	0.46	**3381.7**	0.83	3384.1
wristwatch right now						
c. I am a Verizon customer	0.5	3755.1	0.45	**3675.8**	1	3683.0
Sensitive target items						
e. I would have a problem working	0.5	3559.1	0.40	3474.3	0.43	**3473.3**
with a colleague who is LGB						
d. I would have a problem working	0.49	3363.4	0.45	**3355.2**	0.38	3356.0
with a colleague who is transsexual						
e. I am an LGBT individual	0.5	3598.1	0.24	**3337.2**	0.29	3338.1

The lowest DIC for each item is highlighted in bold.

**Fig. 9:**
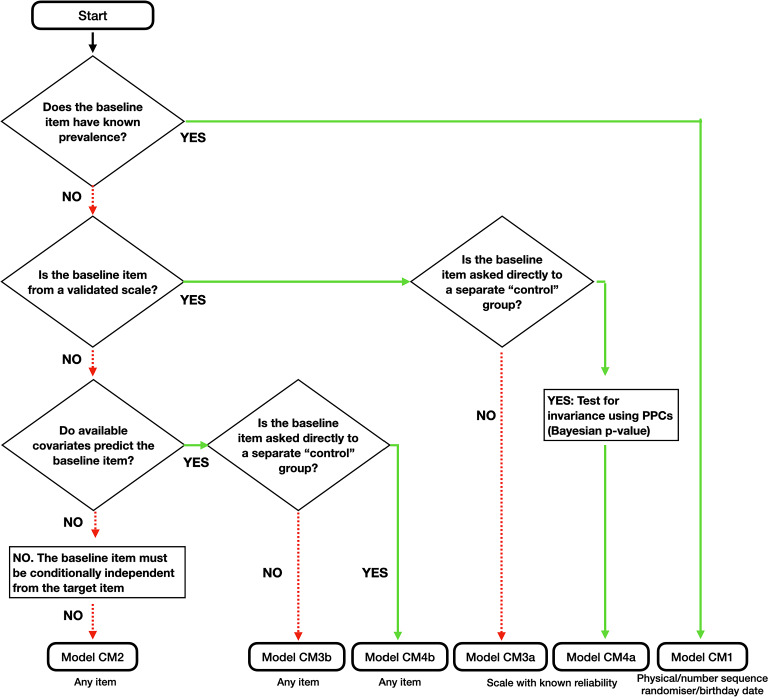
Model selection based on properties of baseline item.

## General Discussion

We provided an integrated methodology for item selection and statistical analysis of the Crosswise Model (CM) to obtain truthful answers to socially sensitive questions. We identified four possible statistical models for analysis and introduced two novel models to predict the baseline item, using IRT and probit regression. The MC simulations and the empirical analyses showed that these novel models can provide higher efficiency than current CM applications that rely on models CM1 and CM2. The novel IRT models can also detect violations of response invariance and statistical independence. DIC supported using either models CM3a or CM4a for all CMs in the empirical application. These statistical models also have more validity at aggregate level using “face validity” criteria, although this approach has been criticized in recent literature (Höglinger and Jann, 2018; Walzenbach and Hinz, 2019). We emphasize that the validity of the model also depends on how well the IRT structure fits the data, and hence, there may be applications where the simpler models CM1 and CM2 outperform empirically the novel models. For reproducibility of our results, and to stimulate further use of the proposed methodology, we provide all code and an interactive app to analyze the data. The flowchart in Fig. 9 aids data analysis based on the selection of baseline item. When using these IRT models, we recommend implementation of Model CM4a with p-groups design, since it is most efficient and allows testing for dependence between the baseline and target item. Nonetheless, appropriate implementation of model CM4a relies on the assumption of response invariance. We thus provide the following guidelines for selection of baseline items and testing:

1. Select scales with moderate-to-low reliability, and nonhomogeneous items. Avoid similarly worded items which might compromise privacy protection and, as a consequence, participants’ trust. For a rule of thumb, we recommend a scale with reliability of .7–.8. If using panels, the outside-the-CM items can also be administered in a separate wave.
2. From the candidate scales, select 2 or at most 3 outside-the-CM items, and 2 or 3 baseline items with expected prevalence between 15 and 35% (or equivalently, between 65 and 85%). A p-groups design is the most efficient choice; however, similar baseline items should not be administered repeatedly. To achieve this, the items can be shuffled around. Multiple scales can also be administered.
3. After data collection, test for invariance based on the PPC. If there is evidence of invariance (i.e., a PPC below .1 or above .9), the DIC can be used to evaluate whether it is best to implement model CM3a in place of model CM4a, trading-off larger variance for smaller bias.
4. We recommend selecting baseline items that are substantively (topic) and semantically (word meaning and form) distant from the target item. For instance, we would not recommend administering target items about academic misconduct with baseline items about conference attendance (as in the study by Jerke et al. (2021)), nor personality traits related to honesty. Distant personality traits may be suitable, such as extraversion and openness to experience. Kuha and Jackson (2014) provides some guidelines for item selection in the context of list experiments which are relevant for applications of the CM as well. Dependence between the target and baseline item in the context of IRT models can be tested using the PPC with models CM3a and CM4a. Parametric dependence between the two items, based on the IRT model, can be modeled as well.

**Table 9:** Applications of different models with other indirect question techniques.

Model	List experiments	Randomized response techniques
Model CM1a	NA	RRT (Imai et al., 2015)
Model CM1b	Single sample count technique	Unrelated question RRT
	(Nepusz et al., 2014)	(Reiber et al., 2020)
Model CM2	Item count technique	Unrelated question RRT
	(Blair and Imai, 2012)	(Kwan et al., 2010)
Model CM3a	Item count response technique	NA
	(De Jong et al., 2010)	
Model CM3b	NA	NA
Model CM4a	NA	NA
Model CM4b	Item count technique	NA
	(Kuha and Jackson, 2014)	

NA, Not applicable (to the best of our knowledge, no data analysis available for the technique).

### Generalization to Other Indirect Question Techniques

Our proposed framework can also applied to other indirect question techniques relying on baseline items, such as List Experiments and some RRTs. RRTs occasionally combine randomization devices with baseline items. For instance, the unrelated question RRT assigns to the survey participants either a target or a baseline item, based on the outcome of a certain randomization device. In the existing literature, this baseline item can either have known prevalence (Model CM1b, as in Reiber et al. (2020)) or can be estimated on the basis of a separate control group (Model CM2, as in Kwan et al. (2010)). In Table 9, we report examples of other indirect question techniques in the literature that leverage any of the models as classified in this research. If the model was not yet proposed, to the best of our knowledge, it is classified as NA. Thus, the framework proposed in this research can be readily extended to different indirect question techniques. This facilitates analysis of pros and cons of baseline items beyond the CM.

In sum, we believe that the simplicity of the CM as compared to alternative indirect question techniques to ask sensitive questions is an important advantage. The CM can be broadly applied in many fields where survey participants may not answer truthfully to sensitive questions, such as health, psychology, economics or marketing. The new statistical models proposed here help to make it reach its potential in terms of providing low estimated variance, and perhaps more validity, for the prevalence of the target item. Statistical checks, such as the DIC test or the test for dependence introduced in this research, may enhance the validity of the resulting estimates. We hope that our research contributes to the more widespread use of the CM to survey sensitive topics and thereby to better theory testing and policy decisions on these topics.

## Supplementary Information

Below is the link to the electronic supplementary material.

Supplementary file 1 (pdf 974 KB)

## Data Availability

Data and materials to replicate the analyses of the research article are available at the following OSF folder: https://osf.io/wprtf/?view_only=c1584740c1b145b9a172b3a82e5ee775.

## Footnotes

1 We adopt the following terminology: we define the technique to collect data in the survey as the “data collection technique”, the statistical model to analyze the data collected as the “statistical model”, and the joint combination of data collection technique and statistical model as “CM methodology”. Also, we use the term “sensitive trait” in a general sense as any position on a state, trait or behavior that the participant considers to be sensitive.

2 As an example, suppose ${{\,\mathrm{\mathbb {P}}\,}}(U_{i} = 1, Z_{i} = 0)=.8$, ${{\,\mathrm{\mathbb {P}}\,}}(U_{i} = 1, Z_{i} = 1)=.6$, and ${{\,\mathrm{\mathbb {P}}\,}}(Z_{i} = 1)=.2$. Then, using Eq. 1, ${{\,\mathrm{\mathbb {P}}\,}}(Y_i = 1)=.72$. Given this observed prevalence, assuming conditional independence of $U_{i}$ and $Z_{i}$ and solving Eq. 3 one obtains an estimated prevalence $\pi =.83$, which overestimates the prevalence of the target item, regardless of the answer to the baseline item $Z_i$.

3 The median sample size of CM studies is $n_\textrm{CM} = 1008$, based on Sagoe et al. (2021). The results are replicated for sample sizes of $n_\textrm{CM} = 500$ and $n_\textrm{CM} = 1500$ in the supplemental material

4 The noninformative prior assigns equal probability to any discrimination parameter between 0 and 4, which is a plausible range for discrimination parameters, given that the latent trait $\theta _i$ is standardized, with mean 0 and variance 1. The informative prior instead assumes that the data generating process for the prior parameters is known. For instance, given the assumed threshold parameters, a reliability of.8 corresponds to drawing parameters $\alpha _{bas}$ from $\log \mathcal {N}(.34,.1)$ (more details are provided in the Web Appendix). We then use as prior in the simulation $\log \mathcal {N}(.34,.1)$.

5 The criterion used here is “more is better” since our sensitive behaviors of interest are assumed to be underreported. The criterion would, however, be more properly called “less is better” if applying the CM with overreported behaviors.
