# Supplementary material for: The Crosswise Model for Surveys on Sensitive Topics: A General Framework for Item Selection and Statistical Analysis
Source: Psychometrika. 2024 May 28;89(3):1007–33. doi: 10.1007/s11336-024-09976-3 (PMC11458659; doi:10.1007/s11336-024-09976-3)
Supplement: Supplementary file 1 — (pdf 974 KB) [file 11336_2024_9976_MOESM1_ESM.pdf]

Supplemental material for: The Crosswise Model for Surveys on Sensitive Topics: A General Framework for Item Selection and Statistical Analysis

The supplemental materials contains:

1. Supplemental material A: Technical Appendix
2. Supplemental material B: Monte Carlo Simulations
3. Supplemental material C: Matlab and Python codes
4. Supplemental material D: Interactive app for the CM
5. Supplemental material E: Suggested items to implement the CM with Model CM3/CM4a
6. Supplemental material F: Additional empirical analyses
7. Supplemental material G: sample size and prevalence of baseline item for past studies

A Technical appendix

Choice of priors

For models CM1 and CM2, the following non-informative prior is used:

$$\mathbb{P}(U_i = 1) \sim \text{Beta}(1, 1)$$
$$\mathbb{P}(Z_i = 1) \sim \text{Beta}(1, 1)$$

In addition, for models CM3a and CM4a, the following priors are used, adapted from Fox, 2010 and De Jong and Pieters, 2019:

$$\alpha_h \sim \text{Uniform}(0, 4)$$
$$\gamma_{h,1} \sim \mathcal{N}(-1, 2)$$
$$\gamma_{h,c} - \gamma_{h,c-1} \sim \log \mathcal{N}(0, 1), \quad \text{where } c > 1$$
$$\alpha_{bas} \sim \text{Uniform}(0, 4)$$
$$\gamma_{bas} \sim \mathcal{N}(0, 1)$$
$$\theta_i \sim \mathcal{N}(0, 1)$$

When including covariates, we adopt a shrinkage prior on the coefficient $\boldsymbol{\beta} \sim \mathcal{N}(0, \lambda\mathbf{I})$, where λ is an analyst-specified shrinkage parameter.

MCMC schemes to estimate models

MCMC sampler for models CM1/CM2. The likelihood of the model is:

$$\mathcal{L}(\mathbf{y}) = \prod_{i=1}^{n_{CM}} \prod_{c \in \{0,1\}} \mathbb{P}(Y_i = c | Z_i, U_i)^{1[Y_i = c]}$$

with $\mathbb{P}(Y_i = 1|Z_i, U_i) = \mathbb{P}(Z_i = 1)\,\mathbb{P}(U_i = 0) + \mathbb{P}(Z_i = 0)\,\mathbb{P}(U_i = 1)$.

1. *Sample* $\mathbb{P}(Z_i = 1)|Z_i$

With model CM1, $\mathbb{P}(Z_i = 1) = \delta$, where δ is a known parameter. With model CM2, $\mathbb{P}(Z_i = 1)$ is not known, but estimated based on a separate group of individuals $i \in 1, \cdots, n_{DQ}$. Hence, we use both the CM group with n_{CM} participants and the separate DQ group with n_{DQ} participants for estimation. Given a Beta$(1, 1)$ prior, the posterior distribution of $\delta = \mathbb{P}(Z_i = 1)$ is a Beta distribution, with the following parameters:

$$\delta \sim \text{Beta}(\sum_{i=1}^{n_{DQ}} Z_i + 1, n_{DQ} - \sum_{i=1}^{n_{DQ}} Z_i + 1)$$

2. *Sample* $\mathbb{P}(Y_i = 1)|Y_i$

Similarly, the posterior distribution of $\mathbb{P}(Y_i = 1)$ is:

$$\mathbb{P}(Y_i = 1) \sim \text{Beta}(\sum_{i=1}^{n_{CM}} Y_i + 1, n_{CM} - \sum_{i=1}^{n_{CM}} Y_i + 1)$$

3. *Sample* $\mathbb{P}(U_i = 1)|Y_i, \delta$

The posterior of $\mathbb{P}(U_i = 1)$ is then computed as:

$$\mathbb{P}(U_i = 1) = \frac{\mathbb{P}(Y_i = 1) - \delta}{1 - 2\delta}$$

Modified MCMC sampler for predicting target item. Covariates can be used to predict the prevalence of the sensitive behavior,:

$$\mathbb{P}(U_i = 1) = \Phi(\beta_0 + \boldsymbol{\beta}_X X_i)$$

For this case, replace step 2. and 3. by the following. We use a Metropolis Hastings step to draw $\boldsymbol{\beta} = \{\beta_0, \boldsymbol{\beta}_X\}$. The candidate $\boldsymbol{\beta}^*$ is generated by a normal distribution:

$$\boldsymbol{\beta}^* \sim \mathcal{N}(\boldsymbol{\beta}^{(m-1)}, \sigma^2_{\boldsymbol{\beta}^*})$$

where $\boldsymbol{\beta}^{(m-1)}$ is the previous draw. The posterior evaluated at the candidate and at the previous draw is proportional to:

$$\prod_{i=1}^{n_{CM}} \delta \times (1 - \Phi(\beta_0 + \boldsymbol{\beta}_X X_i)) + (1 - \delta) \times \Phi(\beta_0 + \boldsymbol{\beta}_X X_i)$$

MCMC sampler for model CM3a

The likelihood of the model is:

$$\mathcal{L}(Y_i, S_{ih}) = \int_\Theta \mathbb{P}(Y_i = 1|\theta_i, \alpha_{bas}, \gamma_{bas}, \pi)^{Y_i}(1 - \mathbb{P}(Y_i = 1|\theta_i, \alpha_{bas}, \gamma_{bas}, \pi))^{1-Y_i}$$
$$\left[\prod_{h=1}^{H}\prod_{c=1}^{C} \mathbb{P}(S_{ih} = c|\theta_i, \alpha_h, \boldsymbol{\gamma}_h)^{1[S_{ih}=c]}\right] \phi(\theta_i|\mu, \sigma)d\theta$$

1a. *Sample* $w_{ih}|S_{ih}, \theta_i, \alpha_h, \boldsymbol{\gamma}_h$ and $w_{i,bas}|Z_i, \theta_i, \alpha_{bas}, \gamma_{bas}$ (data augmentation)

Data augmentation is used for the ordinal and binary responses S_{ih} and Z_i, introducing variables w_{ih} and $w_{i,bas}$ with:

$$w_{ih}|S_{ih}, \theta_i, \alpha_h, \boldsymbol{\gamma}_h \sim \mathcal{N}(\alpha_h \theta_i, 1)\mathbb{1}[\gamma_{h,c-1} < w_{i,bas} < \gamma_{h,c}] \text{ if } S_{ih} = c$$

$$w_{i,bas}|Z_i, \theta_i, \alpha_{bas}, \gamma_{bas} \sim \begin{cases} \mathcal{N}(\alpha_{bas}\theta_i - \gamma_{bas}, 1)\mathbb{1}[w_{i,bas} > 0] \text{ if } Z_i = 1 \\ \mathcal{N}(\alpha_{bas}\theta_i - \gamma_{bas}, 1)\mathbb{1}[w_{i,bas} < 0] \text{ if } Z_i = 0 \end{cases}$$

1b. *Sample* $\theta_i|w_{ih}, w_{i,bas}, \alpha_h, \alpha_{bas}, \gamma_{bas}$ (data augmentation)

Define the vector $\boldsymbol{\alpha} = (\alpha_1, ...\alpha_H, \alpha_{bas})$ and the vector $\mathbf{w}_i = (w_{i,1}, ..., w_{i,H}, w_{i,bas} + \gamma_{bas})$. Given the prior $\phi(\theta_i|0, 1)$, the conditional distribution of the latent trait θ_i is:

$$\theta_i|\boldsymbol{\alpha}, \mathbf{w}_i \sim \mathcal{N}((\boldsymbol{\alpha}'\boldsymbol{\alpha} + 1)^{-1}\boldsymbol{\alpha}'\mathbf{w}_i, (\boldsymbol{\alpha}'\boldsymbol{\alpha} + 1)^{-1})$$

2. *Sample* $\alpha_h|w_{ih}, \theta_i, \boldsymbol{\gamma}_h$ and $\alpha_{bas}|w_{i,bas}, \theta_i, \gamma_{bas}$ (MH step)

The candidate α_h^* is sampled with a Metropolis-Hastings step by a truncated Gaussian distribution:

$$\alpha_h^* \sim \mathcal{N}(\alpha_h^{(m-1)}, \sigma^2_{\alpha_h^*})\mathbb{1}[\alpha_h^* > 0]$$

The posterior evaluated at the candidate and at the previous draw is:

$$\text{Uniform}(\alpha_h|0, 4) \prod_i \phi(w_{ih}|\alpha_h, \boldsymbol{\gamma}_h, \theta_i)$$

For sampling the baseline discrimination parameter α_{bas} the posterior is given by:

$$\text{Uniform}(\alpha_{bas}|0, 4) \prod_i \phi(w_{i,bas}|\alpha_{bas}, \gamma_{bas}, \theta_i)$$

3. *Sample* $\gamma_{h,c}|X_{ih}, \theta_i, \alpha_h$ and $\gamma_{bas}|Z_i, \theta_i, \alpha_{bas}$ (MH step)

For parameter $\gamma_{h,c}$ a truncated normal density is used as proposal:

$$\gamma_{h,c}^* \sim \mathcal{N}(\gamma_{h,c}^{(m-1)}, \sigma^2_{\gamma_h^*})\mathbb{1}[\gamma_{h,c-1}^{(m)} < \gamma_{h,c}^* < \gamma_{h,c+1}^{(m-1)}]$$

Following Fox (2010, chpt. 4), sample a uniform random number u on the interval $[0, 1]$ and

accept the candidate if

$$u \leq \min[\prod_i \frac{\Phi(\alpha_h\theta_i - \gamma^*_{h,c-1}) - \Phi(\alpha_h\theta_i - \gamma^*_{h,c})}{\Phi(\alpha_h\theta_i - \gamma^{(m-1)}_{h,c-1}) - \Phi(\alpha_h\theta_i - \gamma^{(m-1)}_{h,c-1})}$$
$$\prod_{l=1}^{C-1} \frac{\Phi(\frac{\gamma^*_{h,c+1} - \gamma^*_{h,c}}{\sigma_{\gamma^*_h}}) - \Phi(\frac{\gamma^{(m-1)}_{h,c-1} - \gamma^*_{h,c}}{\sigma_{\gamma^*_h}})}{\Phi(\frac{\gamma^{(m)}_{h,c+1} - \gamma^{(m)}_{h,c}}{\sigma_{\gamma^*_h}}) - \Phi(\frac{\gamma^*_{h,c-1} - \gamma^{(m-1)}_{h,c}}{\sigma_{\gamma^*_h}})}, 1]$$

For γ_{bas} a Gaussian distribution is used as proposal:

$$\gamma^*_{bas} \sim \mathcal{N}(\gamma^{(m-1)}_{bas}, \sigma^2_{\gamma^*_{bas}})$$

The posterior evaluated at the candidate and at the previous draw:

$$\phi(\gamma_{bas}|0,1) \prod_i \phi(w_{i,bas}|\alpha_{bas}, \gamma_{bas}, \theta_i)$$

4. *Sample* $U_i|Y_i, \pi, \theta_i, \alpha_{bas}, \gamma_{bas}$ (data augmentation)

Given the unconditional probability $\mathbb{P}(U_i = 1) = \pi$ of affirming the target item U_i, the posterior probabilities conditional on the observe response to the CM Y_i are given by:

$$\mathbb{P}(U_i = 1|Y_i = 1, \alpha_{bas}, \gamma_{bas}, \theta_i) = \frac{\mathbb{P}(U_i = 1)\,\mathbb{P}(Z_i = 0|\theta_i, \alpha_{bas}, \gamma_{bas})}{\mathbb{P}(Y_i = 1|\pi, \theta_i, \alpha_{bas}, \gamma_{bas})}$$
$$\mathbb{P}(U_i = 1|Y_i = 0, \alpha_{bas}, \gamma_{bas}, \theta_i) = \frac{\mathbb{P}(U_i = 1)\,\mathbb{P}(Z_i = 1|\theta_i, \alpha_{bas}, \gamma_{bas})}{\mathbb{P}(Y_i = 0|\pi, \theta_i, \alpha_{bas}, \gamma_{bas})}$$

The posterior probability is then:

$$\mathbb{P}(U_i = 1) = Y_i \times \mathbb{P}(U_i = 1|Y_i = 1, \alpha_{bas}, \gamma_{bas}, \theta_i) + (1 - Y_i) \times \mathbb{P}(U_i = 1|Y_i = 0, \alpha_{bas}, \gamma_{bas}, \theta_i)$$

Use these probabilities to sample latent dummy variables U_i.

5. *Sample* $Z_i|Y_i, \theta_i, \alpha_{bas}, \gamma_{bas}$ (data augmentation)

The posterior probabilities for the baseline items are similar to step 4.

6. *Sample* $\pi|U_i$

The draws for π are given by a Beta distribution, with the following parameters:

$$\pi \sim \text{Beta}(\sum_{i=1}^{n_{CM}} U_i + 1, n_{CM} - \sum_{i=1}^{n_{CM}} U_i + 1)$$

Modified MCMC scheme for predicting target item. Covariates $\mathbf{x}_i$ can be used to predict the prevalence of the sensitive behavior as well as to relate such prevalence to the latent trait θ_i:

$$\mathbb{P}(U_i = 1) = \Phi(\beta_0 + \theta_i\beta_\theta + \mathbf{x}_i\boldsymbol{\beta}_X)$$

Using this model extension, we rely on the outside-the-pair items to estimate both the coefficient β_θ as well as the answers to the baseline item. Thus, the empirical identification is *frail.* However, we can better identify the coefficient β_θ by adding more outside-the-pair items S_{ih} or using p-groups design. We only give the sampler for model CM3, as the extension for model CM4a is trivial. Let $\boldsymbol{\beta} = \{\beta_0, \beta_\theta, \boldsymbol{\beta}_X\}$. To sample these parameters, replace step 1b. and 8. by the following:

1b. *Sample* $\theta_i|w_{ih}, w_{i,bas}, , \alpha_h, \alpha_{bas}, \gamma_{bas}, \boldsymbol{\beta}$ (data augmentation)

Define the vector $\boldsymbol{\alpha} = \{\alpha_1, \cdots, \alpha_H, \alpha_{bas}, \beta_\theta\}$, and the vector $\mathbf{w}_i = \{w_{i,1}, \cdots, w_{i,H}, w_{i,bas} + \gamma_{bas}, w_{i,u} - \beta_0 - \mathbf{x}_i\boldsymbol{\beta}_X\}$, where $w_{i,u}$ is sampled in step 8a. Given the prior $\phi(\theta_i|0,1)$, sample the latent traits θ_i as

$$\theta_i|\boldsymbol{\alpha}, \mathbf{w}_i \sim \mathcal{N}((\boldsymbol{\alpha}'\boldsymbol{\alpha} + 1)^{-1}\boldsymbol{\alpha}'\mathbf{w}_i, (\boldsymbol{\alpha}'\boldsymbol{\alpha} + 1)^{-1})$$

8a. *Sample* $w_{i,u}|\boldsymbol{\beta}, \mathbf{x}_i, \theta_i$ (data augmentation)

Data augmentation is used, with the following conditional distribution:

$$w_{i,u}|\boldsymbol{\beta}, \mathbf{x}_i \sim \begin{cases} \Phi(\beta_0 + \theta_i\beta_\theta + \mathbf{x}_i\boldsymbol{\beta}_X)\mathbb{1}[w_{i,u} > 0] \text{ if } U_i = 1 \\ \Phi(\beta_0 + \theta_i\beta_\theta + \mathbf{x}_i\boldsymbol{\beta}_X)\mathbb{1}[w_{i,u} < 0] \text{ if } U_i = 0 \end{cases}$$

8b. *Sample* $\boldsymbol{\beta}|\mathbf{w}_u, \mathbf{x}_i, \theta_i$

Denote the N by $K+1$ matrix $\mathbf{X}$ containing covariates $\mathbf{x}_i$ and the vector of latent traits θ_i, such that $\mathbf{X}_i = \{1, \theta_i, \mathbf{x}_i\}$. Sample the coefficients $\boldsymbol{\beta}$ as:

$$\boldsymbol{\beta}|\mathbf{X}, \mathbf{w}_u \sim \mathcal{N}((\mathbf{X}'\mathbf{X} + \lambda^{-1}\mathbf{I}_{K+1})^{-1}\mathbf{X}'\mathbf{w}_u, (\mathbf{X}'\mathbf{X} + \lambda^{-1}\mathbf{I}_{K+1})^{-1})$$

When using a p-groups design with model CM3a $w_{i,bas}$ is missing if the baseline item is not administered in the PRT. To obviate this, the missing $w_{i,bas}$ are simulated as $w^*_{i,bas} \sim \mathcal{N}(\alpha_{bas}\theta_i - \gamma_{bas}, 1)$.

Modified MCMC sampler for model CM4a

With model CM4a, we use both the CM group with n_{CM} participants and the separate DQ group with n_{DQ} participants. In the CM group, the latent response $Z_i^{(CM)}$ is not observed and in the DQ group the response $Z_i^{(DQ)}$ is observed. CM4a then uses the same sampler as model CM3a. There are two key differences:

- A separate direct question group is asked the baseline item, as with model CM2. The answers to the outside-the-CM items and the (direct) answers to the baseline item in this group are also used to estimate α_h, α_{bas}, $\boldsymbol{\gamma}_h$ and γ_{bas}.
- For the separate direct question group we do not observe the latent answers to the target item, because the CM is not administered. Therefore, we simulate the missing values $w_{i,u}$ from the remaining model parameters (if required for step 8)

MCMC sampler for model CM3b

The equation to predict the baseline item with model CM3b is:

$$\mathbb{P}(Z_i = 1 | \mathbf{x}_i, \boldsymbol{\delta}_{bas}) = \Phi(\delta_0 + \mathbf{x}_i \boldsymbol{\delta}_{bas})$$

1. *Sample* $w_{i,z} | \boldsymbol{\delta}_{bas}, \mathbf{x}_i$ (data augmentation)

Data augmentation is used, with the following conditional distribution:

$$w_{i,z} | \boldsymbol{\delta}_{bas}, \mathbf{x}_i \sim \begin{cases} \Phi(\delta_0 + \mathbf{x}_i \boldsymbol{\delta}_{bas}) \mathbb{1}[w_{i,z} > 0] \text{ if } Z_i = 1 \\ \Phi(\delta_0 + \mathbf{x}_i \boldsymbol{\delta}_{bas}) \mathbb{1}[w_{i,z} < 0] \text{ if } Z_i = 0 \end{cases}$$

2. *Sample* $\boldsymbol{\delta}_{bas} | \mathbf{X}, \mathbf{w}_z$

We assume the N by K matrix $\mathbf{X}$ include a column of dummies. Sample the K coefficients $\{\delta_0, \boldsymbol{\delta}_{bas}\}$ as:

$$\{\delta_0, \boldsymbol{\delta}_{bas}\} | \mathbf{X}, \mathbf{w}_z \sim \mathcal{N}((\mathbf{X}'\mathbf{X} + \lambda^{-1}\mathbf{I}_K)^{-1}\mathbf{X}'\mathbf{w}_z, (\mathbf{X}'\mathbf{X} + \lambda^{-1}\mathbf{I}_K)^{-1})$$

3. *Sample* $Z_i|Y_i, \pi, \mathbf{x}_i, \delta_0, \boldsymbol{\delta}_{bas}$ (data augmentation)

The latent dummies Z_i are sampled using data augmentation. The posterior probabilities for the target items Z_i are given by:

$$\mathbb{P}(Z_i = 1|Y_i = 1) = \frac{\mathbb{P}(U_i = 0)\,\mathbb{P}(Z_i = 1|\mathbf{x}_i, \delta_0, \boldsymbol{\delta}_{bas})}{\mathbb{P}(Y_i = 1|\pi, \mathbf{x}_i, \delta_0, \boldsymbol{\delta}_{bas})}$$
$$\mathbb{P}(Z_i = 1|Y_i = 0) = \frac{\mathbb{P}(U_i = 1)\,\mathbb{P}(Z_i = 1|\mathbf{x}_i, \delta_0, \boldsymbol{\delta}_{bas})}{\mathbb{P}(Y_i = 0|\pi, \mathbf{x}_i, \delta_0, \boldsymbol{\delta}_{bas})}$$

Use these probabilities to draw dummies for Z_i.

4. *Sample* $U_i|Y_i, \pi, \mathbf{x}_i, \delta_0, \boldsymbol{\delta}_{bas}$ (data augmentation)

The posterior probabilities for the baseline items U_i are similar to step 3. These are sampled using data augmentation only for the CM group.

5. *Sample* $\pi|U_i$

The posterior for $\pi = \mathbb{P}(U_i = 1)$ is given by a Beta distribution, with the following parameters:

$$\pi \sim \text{Beta}(\sum_{i=1}^{n_{CM}} U_i + 1, n_{CM} - \sum_{i=1}^{n_{CM}} U_i + 1)$$

Modified MCMC sampler for model CM4b

With model CM4b, we use both the CM group with n_{CM} participants and the separate DQ group with n_{DQ} participants. In the CM group, the latent response $Z_i^{(CM)}$ is not observed and in the DQ group the response $Z_i^{(DQ)}$ is observed. CM4b then uses the same sampler as model CM3b (only $Z_i^{(DQ)}$ are not sampled at step 3.).

B MC simulations

Here we provide more details on the three MC simulations illustrated in the main paper.

B.1 First MC simulation: comparing model CM1 vs. blocked design of model CM4a and CM4b

In this first MC simulation, we report the simulations outcomes when implementing models CM1, CM4a and CM4b. For both models CM4a and CM4b we assume a 2-groups design. Each baseline

item has symmetric prevalence (for instance, 90% and 10%, or 80% and 20%). For the purpose of accuracy and MSE, the end results are the same if, say, the prevalence of the baseline item is 80 % or 20 %.

For model CM4a the following model parameters were used: for the ordered Probit model with latent trait $\theta_i \sim \mathcal{N}(0, 1)$ in equation 4, we draw threshold parameters as follows. The first threshold parameter is simulated as: $\gamma_{h,1} \sim$ Uniform$(-1, .5)$. The next threshold parameters are then simulated as: $\gamma_{h,c} - \gamma_{h,c-1} \sim$ Uniform$(.2, 1)$. We then simulate discrimination parameters $\alpha_h, \alpha_{bas} \sim \log\mathcal{N}(\bar{\alpha}, .1)$, where we vary the parameter $\bar{\alpha}$ to obtain a certain scale reliability (Cronbach alpha). That is, to obtain reliability .8, we set $\bar{\alpha} = .34$, to obtain reliability .7 $\bar{\alpha} = .05$, and to obtain reliability .6 $\bar{\alpha} = -.22$. The different reliability values are reported in the rows of Tables S1 to S3. The threshold parameter of the baseline item are varied as $\gamma_{bas} \in \{1.5, 1.25, 1, .75, .5\}$ in one group and as $\gamma_{bas} \in \{-1.5, -1.25, -1, -.75, -.5\}$ in the other group. These are reported in the columns of Tables S2 to S10. The sample size are varied as $n_{CM} \in \{500, 1000, 1500\}$ and the prevalences of the target item are varied as $\pi \in \{10\%, 20\%, 30\%\}$. Model CM1 is then fit to the same data.

For model CM4b, we assume a Probit model with intercept $\delta_0 \in \{1.5, 1.25, 1, .75, .5\}$ ($\{-1.5, -1.25, -1, -.75, -.$[illegible] and a single explanatory variable x_i with coefficient $\delta_{bas} \in \{.33, .5, .66\}$ in equation 8. This correspond to a pseudo $R^2 \in \{.1, .2, .3\}$. For each model, we use 10000 draws for burnin and 20000 draws for estimation. 200 simulations were implemented for each scenario using the Matlab codes. Codes for replication for each model are included in the .m files *MC1_ModelCM4a.m* and *MC1_ModelCM4b.m*. The metrics used for evaluation are:

- Average estimated prevalence of the target item across S simulations (as a measure of accuracy): if the estimated mean in simulation s is $\hat{\pi}_s$, we report $\sum_{s=1}^{S} \hat{\pi}_s$
- Average estimated posterior standard error of the prevalence of the target item (as a measure of efficiency). The estimated standard error is estimated from the posterior draws. If the estimated standard error in simulation s is $\hat{\sigma}_{\pi,s}$, we report $\sum_{s=1}^{S} \hat{\sigma}_{\pi,s}$
- Average Mean Squared Error (MSE) across S simulations. Given the true prevalence π, this

is computed as:

$$MSE = \sum_{s=1}^{S} \hat{\sigma}^2_{\pi,s} + (\hat{\pi}_s - \pi)^2$$

In the following tables we report the estimated means (Tables S1 and S4), estimated variances (Tables S2 and S5) and Mean Squared Error (Tables S3 and S6). It is then easy to see that:

- All 3 models correctly estimate the average prevalence of the target item
- For most combination of parameters models CM4a and CM4b have lower variance and lower MSE as compared to model CM1. Model CM1 can outperform either model when there is limited information to predict the baseline item, for instance, when the scale reliability is less than .6 or when the pseudo-R^2 is .1 or less.

B.2 Second MC simulation: models CM3a

For the second set of MC simulations the data generating process is as in the first set of MC simulations. However, we focus on the case $\gamma_{bas} = -1$ (this corresponds approximately to the median prevalence of the baseline item in published CM studies, as inferred from Sagoe et al. (2021)). We examine two separate implementations of model CM3a: one with non-informative prior for the discrimination parameter ($\alpha_{bas} \sim$ Uniform$(0, 4)$) and one with informative priors, based on the scale reliability ($\log \mathcal{N}(\bar{\alpha}, .1)$, with $\bar{\alpha} \in \{-.22, .05, .34\}$). $\bar{\alpha}$ is varied as described in the previous set of simulations and assumed to be known (e.g., because of external knowledge on the scale reliability or from a separate sample). As in the previous sets of simulations, we report on results with samples sizes $n_{CM} \in \{500, 1000, 1500\}$. Both models seem suitable to estimate the prevalence of the target item, although performance worsens with smaller sample sizes or when the scale reliability/pseudo-R^2 is low. The results suggest that sample sizes above 1000 survey participants are recommended when implementing models CM3a and CM3b. Codes for replication are included in the .m files *MC2_M3a_inf.m*, *MC2_M3a_noninf.m* and *MC2_M3b.m*.

B.3 Third MC simulation: violating invariance with models CM3a and CM4a

In the third MC simulation the data generating process is as in the first set of MC simulations. However, we assume reliability of either .7 or .8 ($\bar{\alpha} \in \{.05, .34\}$)) and $\gamma_{bas} = -1$. We then gradually

Table S1: MC simulation 1: Estimated Means (in Percentage) for CM4a and CM1

		Model CM4a					Model CM1				
	Scale reliability										
N = 500	π = 10 %	±1.5	±1.25	±1	±0.75	±0.5	±1.5	±1.25	±1	±0.75	±0.5
	0.6	10.7	10.5	11.3	11.7	11.5	10.7	10.2	10.8	10.7	9.4
	0.7	10.5	10.5	10.5	11.0	11.2	10.5	10.3	10.5	10.7	9.4
	0.8	10.4	10.3	10.7	10.7	10.8	10.7	10.3	10.3	10.9	9.1
	π = 20 %										
	0.6	20.4	20.4	20.5	20.6	21.6	20.5	20.4	20.1	19.7	20.5
	0.7	20.4	20.4	20.5	20.8	21.0	20.6	20.5	20.1	20.5	20.4
	0.8	20.3	20.3	20.4	20.7	20.8	20.5	19.8	20.1	20.2	22.4
	π = 30 %										
	0.6	30.4	29.9	30.8	30.6	30.7	30.7	29.9	30.6	30.2	29.5
	0.7	30.4	30.2	30.3	30.4	31.0	30.5	30.3	30.5	30.2	30.7
	0.8	30.3	30.2	30.0	30.7	30.2	30.7	30.1	30.2	30.5	29.3
N = 1000	π = 10 %										
	0.6	10.2	10.1	10.4	10.5	10.9	10.2	10.0	10.1	10.1	10.3
	0.7	10.1	10.1	10.2	10.4	10.2	10.1	10.1	10.2	9.9	10.5
	0.8	10.1	10.2	10.3	10.2	10.4	10.0	10.4	10.3	9.9	10.1
	π = 20 %										
	0.6	20.3	20.0	20.2	20.2	21.0	20.2	19.9	20.1	19.8	20.8
	0.7	20.2	19.9	20.0	20.4	20.3	20.1	19.9	20.0	20.2	20.5
	0.8	20.2	20.0	20.0	20.3	20.1	20.1	19.8	19.9	20.1	20.7
	π = 30 %										
	0.6	30.0	29.8	29.8	30.2	30.2	30.0	29.8	29.9	30.3	29.7
	0.7	30.0	29.7	29.8	30.2	30.3	30.0	29.8	30.0	30.4	30.4
	0.8	30.2	30.2	30.0	30.3	30.4	30.2	30.3	30.1	30.4	30.9
N = 1500	π = 10 %										
	0.6	10.4	10.1	10.2	10.5	10.6	10.3	10.1	10.0	10.2	9.9
	0.7	10.3	10.0	10.3	10.3	10.6	10.4	10.1	10.1	10.1	10.5
	0.8	10.3	10.1	10.2	10.5	10.6	10.3	9.9	10.0	10.5	10.6
	π = 20 %										
	0.6	20.3	20.2	20.2	20.4	20.4	20.3	20.1	20.1	20.2	19.6
	0.7	20.3	20.2	20.1	20.4	20.1	20.3	20.2	20.0	20.1	19.8
	0.8	20.4	20.2	20.3	20.4	20.2	20.3	20.2	20.1	20.2	19.8
	π = 30 %										
	0.6	30.3	30.0	30.5	30.3	29.7	30.2	30.0	30.3	30.1	29.3
	0.7	30.2	29.9	30.5	30.3	29.8	30.2	30.1	30.3	29.9	29.5
	0.8	30.3	30.2	30.1	30.3	30.0	30.2	30.4	30.1	29.8	30.7

Table S2: MC simulation 1: Estimated Variances for CM4a and CM1

		Model CM4a					Model CM1				
	Scale reliability										
N = 500	π = 10 %	±1.5	±1.25	±1	±0.75	±0.5	±1.5	±1.25	±1	±0.75	±0.5
	0.6	5.4	7.0	9.9	13.9	19.2	5.6	8.0	12.7	23.2	54.3
	0.7	5.4	6.6	8.5	11.3	13.8	7.1	10.4	16.6	30.2	74.3
	0.8	5.1	5.8	7.0	8.1	9.2	10.2	14.8	24.2	43.9	109.2
	π = 20 %										
	0.6	6.8	8.6	11.5	15.4	22.2	6.9	9.3	14.1	23.7	55.3
	0.7	7.1	8.6	10.6	13.5	16.7	8.5	11.7	17.8	31.1	74.3
	0.8	7.1	8.2	9.2	10.5	11.6	11.6	16.4	25.5	44.3	109.1
	π = 30 %										
	0.6	7.8	9.4	12.1	16.2	22.0	7.9	10.3	14.9	25.5	56.1
	0.7	8.1	9.7	11.7	14.1	17.2	9.5	12.6	18.7	32.5	73.8
	0.8	8.3	9.3	10.3	11.3	12.3	12.6	17.1	26.1	46.8	114.3
N = 1000	π = 10 %										
	0.6	2.7	3.6	5.2	7.6	11.2	2.8	4.0	6.3	11.3	26.0
	0.7	2.7	3.4	4.4	5.8	7.7	3.5	5.1	8.1	14.7	34.0
	0.8	2.5	3.0	3.5	4.1	4.8	5.0	7.3	11.7	21.3	48.8
	π = 20 %										
	0.6	3.4	4.3	5.9	8.2	11.9	3.4	4.6	7.0	12.0	27.0
	0.7	3.5	4.3	5.4	6.8	8.5	4.2	5.8	8.8	15.6	34.3
	0.8	3.5	4.0	4.6	5.2	5.8	5.8	7.9	12.2	22.1	49.1
	π = 30 %										
	0.6	3.8	4.7	6.2	8.4	11.6	3.9	5.2	7.5	12.7	27.3
	0.7	4.0	4.9	5.9	7.2	8.6	4.7	6.4	9.3	16.1	35.4
	0.8	4.1	4.6	5.2	5.7	6.2	6.2	8.6	12.9	22.4	50.7
N = 1500	π = 10 %										
	0.6	1.8	2.5	3.5	5.2	7.9	1.9	2.6	4.2	7.7	17.6
	0.7	1.8	2.3	3.0	4.0	5.1	2.4	3.4	5.4	9.9	22.8
	0.8	1.7	1.9	2.4	2.8	3.2	3.3	4.9	7.7	14.2	33.1
	π = 20 %										
	0.6	2.3	2.9	3.9	5.7	8.0	2.3	3.1	4.6	8.0	18.0
	0.7	2.4	2.9	3.6	4.6	5.7	2.8	3.9	5.9	10.3	23.2
	0.8	2.4	2.7	3.1	3.5	3.9	3.8	5.4	8.4	14.5	33.0
	π = 30 %										
	0.6	2.5	3.2	4.1	5.6	7.8	2.6	3.4	5.0	8.4	18.2
	0.7	2.7	3.2	3.9	4.8	5.8	3.2	4.2	6.3	10.7	23.5
	0.8	2.8	3.1	3.5	3.8	4.2	4.2	5.7	8.6	14.7	33.2

Table S3: MC simulation 1: Mean Squared Error for CM4a and CM1

		Model CM4a					Model CM1				
	Scale reliability										
N = 500	π = 10 %	±1.5	±1.25	±1	±0.75	±0.5	±1.5	±1.25	±1	±0.75	±0.5
	0.1	4.3	6.0	9.2	14.1	23.3	3.8	5.3	8.4	15.0	36.1
	0.2	4.2	5.7	8.3	12.2	19.1	4.2	6.0	9.6	17.2	40.4
	0.3	4.1	5.4	7.4	10.3	14.0	4.8	6.9	11.0	19.8	46.9
	π = 20 %										
	0.1	5.5	7.2	10.5	16.8	29.2	5.1	6.7	9.8	16.5	36.8
	0.2	5.6	7.2	9.9	14.4	22.7	5.5	7.4	11.0	18.4	42.8
	0.3	5.7	7.1	9.3	12.6	17.4	6.1	8.3	12.4	21.4	47.8
	π = 30 %										
	0.1	6.3	7.9	10.8	16.3	29.4	6.1	7.7	10.8	17.4	38.1
	0.2	6.5	8.0	10.5	14.8	22.9	6.5	8.4	12.0	19.4	42.8
	0.3	6.6	8.1	10.1	13.4	18.2	7.1	9.3	13.3	22.1	49.3
N = 1000	π = 10 %										
	0.1	2.2	3.1	4.8	8.0	13.9	1.9	2.7	4.2	7.5	17.3
	0.2	2.2	3.0	4.2	6.6	10.2	2.1	3.0	4.7	8.5	19.4
	0.3	2.1	2.8	3.8	5.4	7.6	2.4	3.5	5.5	9.9	22.4
	π = 20 %										
	0.1	2.8	3.7	5.2	8.4	15.1	2.6	3.4	4.9	8.2	17.8
	0.2	2.8	3.6	4.9	7.2	11.3	2.8	3.7	5.4	9.2	20.1
	0.3	2.9	3.6	4.6	6.3	8.8	3.1	4.2	6.1	10.5	23.3
	π = 30 %										
	0.1	3.2	4.0	5.4	8.2	14.3	3.1	3.9	5.4	8.7	18.2
	0.2	3.3	4.0	5.2	7.5	11.2	3.3	4.2	5.9	9.7	20.7
	0.3	3.4	4.1	5.1	6.7	9.0	3.6	4.7	6.6	11.0	23.7
N = 1500	π = 10 %										
	0.1	1.5	2.1	3.2	5.5	9.9	1.3	1.8	2.8	5.0	11.5
	0.2	1.4	1.9	2.8	4.4	7.2	1.4	2.0	3.1	5.7	13.0
	0.3	1.4	1.8	2.5	3.6	5.2	1.6	2.3	3.6	6.5	15.1
	π = 20 %										
	0.1	1.9	2.4	3.4	5.6	10.2	1.7	2.2	3.2	5.5	12.0
	0.2	1.9	2.4	3.3	4.8	7.6	1.9	2.5	3.6	6.1	13.5
	0.3	1.9	2.4	3.1	4.2	5.8	2.1	2.8	4.1	7.0	15.5
	π = 30 %										
	0.1	2.1	2.6	3.6	5.5	9.6	2.1	2.6	3.6	5.8	12.3
	0.2	2.2	2.7	3.5	5.0	7.5	2.2	2.8	3.9	6.5	13.8
	0.3	2.2	2.7	3.4	4.5	6.0	2.4	3.1	4.4	7.4	15.8

Table S4: MC simulation 1: Estimated Means (in Percentage) for CM4b and CM1

		Model CM4b					Model CM1				
	Pseudo-R^2										
N = 500	π = 10 %	±1.5	±1.25	±1	±0.75	±0.5	±1.5	±1.25	±1	±0.75	±0.5
	0.1	10.1	10.0	10.8	10.7	11.4	10.1	10.1	10.5	10.3	10.3
	0.2	10.2	10.5	10.6	11.0	11.8	10.2	10.4	10.5	11.0	11.3
	0.3	10.2	10.4	10.6	10.8	11.1	10.2	10.3	10.7	10.7	10.4
	π = 20 %										
	0.1	19.9	20.1	20.2	19.8	20.3	19.9	20.1	20.3	20.0	19.8
	0.2	19.9	20.2	20.4	20.2	20.5	19.9	20.2	20.4	20.3	20.1
	0.3	19.9	20.2	20.4	20.0	20.5	19.8	20.1	20.4	20.4	20.2
	π = 30 %										
	0.1	30.0	30.0	30.5	30.5	30.5	30.1	30.1	30.4	30.5	30.7
	0.2	30.1	30.0	30.8	30.0	30.6	30.1	29.9	30.7	30.3	30.9
	0.3	30.1	30.1	30.7	29.9	30.6	30.0	29.9	30.6	29.9	31.5
N = 1000	π = 10 %										
	0.1	10.2	10.4	10.1	9.9	11.1	10.2	10.4	10.1	10.0	10.5
	0.2	10.2	10.4	10.1	10.1	10.6	10.2	10.4	10.1	10.2	10.2
	0.3	10.2	10.3	10.3	10.1	10.4	10.1	10.3	10.3	10.2	10.3
	π = 20 %										
	0.1	20.1	20.4	20.2	20.1	20.1	20.1	20.4	20.2	20.3	20.1
	0.2	20.1	20.4	20.3	20.0	20.0	20.0	20.5	20.3	20.2	20.0
	0.3	20.1	20.4	20.3	20.2	20.1	20.1	20.4	20.2	20.3	20.2
	π = 30 %										
	0.1	30.0	30.3	30.3	30.0	30.2	30.0	30.4	30.4	30.1	30.3
	0.2	30.0	30.3	30.3	30.2	30.2	30.0	30.4	30.3	30.4	30.3
	0.3	30.1	30.3	30.3	30.1	30.2	30.0	30.4	30.3	30.2	30.4
N = 1500	π = 10 %										
	0.1	10.2	10.1	10.2	9.8	10.5	10.2	10.1	10.1	9.8	10.1
	0.2	10.1	10.1	10.1	9.9	10.2	10.2	10.1	10.1	9.7	10.0
	0.3	10.1	10.1	10.1	9.9	10.1	10.2	10.0	10.1	9.6	9.7
	π = 20 %										
	0.1	20.2	19.9	20.2	19.8	20.0	20.2	19.9	20.2	19.9	20.0
	0.2	20.1	20.0	20.0	19.8	20.1	20.2	20.0	20.0	19.7	19.9
	0.3	20.2	19.9	20.2	19.8	20.2	20.2	19.9	20.2	19.6	20.0
	π = 30 %										
	0.1	30.1	30.0	30.1	29.9	30.2	30.2	30.0	30.1	30.0	29.8
	0.2	30.1	29.9	30.0	29.9	30.2	30.2	29.9	30.0	29.8	30.1
	0.3	30.2	29.9	30.0	29.9	30.3	30.2	29.9	30.0	29.8	29.9

Table S5: MC simulation: Estimated Variances for CM4b and CM1

		Model CM4b					Model CM1				
	Pseudo-R^2										
N = 500	$\pi = 10$ %	±1.5	±1.25	±1	±0.75	±0.5	±1.5	±1.25	±1	±0.75	±0.5
	0.1	4.3	6.0	9.2	14.1	23.3	3.8	5.3	8.4	15.0	36.1
	0.2	4.2	5.7	8.3	12.2	19.1	4.2	6.0	9.6	17.2	40.4
	0.3	4.1	5.4	7.4	10.3	14.0	4.8	6.9	11.0	19.8	46.9
	$\pi = 20$ %										
	0.1	5.5	7.2	10.5	16.8	29.2	5.1	6.7	9.8	16.5	36.8
	0.2	5.6	7.2	9.9	14.4	22.7	5.5	7.4	11.0	18.4	42.8
	0.3	5.7	7.1	9.3	12.6	17.4	6.1	8.3	12.4	21.4	47.8
	$\pi = 30$ %										
	0.1	6.3	7.9	10.8	16.3	29.4	6.1	7.7	10.8	17.4	38.1
	0.2	6.5	8.0	10.5	14.8	22.9	6.5	8.4	12.0	19.4	42.8
	0.3	6.6	8.1	10.1	13.4	18.2	7.1	9.3	13.3	22.1	49.3
N = 1000	$\pi = 10$ %										
	0.1	2.2	3.1	4.8	8.0	13.9	1.9	2.7	4.2	7.5	17.3
	0.2	2.2	3.0	4.2	6.6	10.2	2.1	3.0	4.7	8.5	19.4
	0.3	2.1	2.8	3.8	5.4	7.6	2.4	3.5	5.5	9.9	22.4
	$\pi = 20$ %										
	0.1	2.8	3.7	5.2	8.4	15.1	2.6	3.4	4.9	8.2	17.8
	0.2	2.8	3.6	4.9	7.2	11.3	2.8	3.7	5.4	9.2	20.1
	0.3	2.9	3.6	4.6	6.3	8.8	3.1	4.2	6.1	10.5	23.3
	$\pi = 30$ %										
	0.1	3.2	4.0	5.4	8.2	14.3	3.1	3.9	5.4	8.7	18.2
	0.2	3.3	4.0	5.2	7.5	11.2	3.3	4.2	5.9	9.7	20.7
	0.3	3.4	4.1	5.1	6.7	9.0	3.6	4.7	6.6	11.0	23.7
N = 1500	$\pi = 10$ %										
	0.1	1.5	2.1	3.2	5.5	9.9	1.3	1.8	2.8	5.0	11.5
	0.2	1.4	1.9	2.8	4.4	7.2	1.4	2.0	3.1	5.7	13.0
	0.3	1.4	1.8	2.5	3.6	5.2	1.6	2.3	3.6	6.5	15.1
	$\pi = 20$ %										
	0.1	1.9	2.4	3.4	5.6	10.2	1.7	2.2	3.2	5.5	12.0
	0.2	1.9	2.4	3.3	4.8	7.6	1.9	2.5	3.6	6.1	13.5
	0.3	1.9	2.4	3.1	4.2	5.8	2.1	2.8	4.1	7.0	15.5
	$\pi = 30$ %										
	0.1	2.1	2.6	3.6	5.5	9.6	2.1	2.6	3.6	5.8	12.3
	0.2	2.2	2.7	3.5	5.0	7.5	2.2	2.8	3.9	6.5	13.8
	0.3	2.2	2.7	3.4	4.5	6.0	2.4	3.1	4.4	7.4	15.8

Table S6: MC simulation 1: Mean Squared Error for CM4b and CM1

	Pseudo-R^2	Model CM4b					Model CM1				
N = 500	$\pi = 10$ %	±1.5	±1.25	±1	±0.75	±0.5	±1.5	±1.25	±1	±0.75	±0.5
	0.1	9.2	12.3	17.9	27.1	43.3	7.5	9.6	14.8	26.0	61.2
	0.2	8.8	11.6	16.0	24.6	38.2	8.1	10.9	16.9	29.7	70.2
	0.3	8.3	10.8	13.9	19.9	29.7	9.1	12.2	18.8	33.6	82.9
	$\pi = 20$ %										
	0.1	11.2	16.2	21.2	35.3	54.7	10.5	14.2	18.7	30.7	68.3
	0.2	11.0	15.5	20.1	28.6	44.4	10.9	15.1	20.3	33.8	85.4
	0.3	11.5	15.8	17.8	26.1	34.2	11.9	16.7	22.2	42.8	94.9
	$\pi = 30$ %										
	0.1	11.7	16.3	21.0	30.4	55.6	11.5	15.8	21.1	31.8	69.3
	0.2	11.9	16.4	20.7	27.3	46.9	12.2	16.1	23.8	33.3	79.1
	0.3	12.5	16.9	19.0	25.7	38.3	13.4	18.9	24.7	40.3	88.8
N = 1000	$\pi = 10$ %										
	0.1	4.1	6.7	10.6	15.4	29.0	3.3	5.2	7.9	12.9	31.9
	0.2	4.2	6.3	8.8	13.4	20.9	3.8	5.9	8.6	15.6	34.1
	0.3	4.0	5.5	8.3	9.6	16.7	4.4	6.6	9.9	16.8	40.6
	$\pi = 20$ %										
	0.1	5.2	7.6	10.7	16.4	28.6	4.7	6.6	9.0	15.1	29.7
	0.2	5.5	8.1	9.7	14.1	22.1	5.3	7.7	10.2	16.6	35.0
	0.3	5.6	7.5	9.0	12.0	18.9	6.2	8.2	11.4	18.2	45.4
	$\pi = 30$ %										
	0.1	5.8	8.1	11.2	14.7	29.8	5.7	8.0	10.7	14.8	36.1
	0.2	6.0	8.0	10.5	14.5	23.6	6.2	8.6	11.0	17.1	40.2
	0.3	6.1	8.0	10.0	13.0	18.1	6.9	9.3	12.4	20.2	46.5
N = 1500	$\pi = 10$ %										
	0.1	2.7	4.2	6.7	11.4	20.4	2.2	3.2	4.9	9.0	20.1
	0.2	2.7	3.9	5.9	8.8	15.1	2.4	3.5	5.6	9.8	22.7
	0.3	2.5	3.7	5.1	7.3	10.8	2.7	4.2	6.5	11.5	28.4
	$\pi = 20$ %										
	0.1	3.7	5.2	8.0	12.3	19.2	3.5	4.4	6.8	11.0	21.4
	0.2	3.8	5.1	8.2	10.0	15.3	3.7	4.8	8.2	11.7	26.7
	0.3	3.9	5.0	7.2	8.3	12.0	4.1	5.3	8.9	13.3	31.0
	$\pi = 30$ %										
	0.1	4.4	5.5	7.8	10.6	18.1	4.4	5.1	7.7	11.0	23.5
	0.2	4.3	5.6	8.0	9.9	14.6	4.4	5.5	8.7	12.1	25.5
	0.3	4.5	5.7	7.5	9.1	12.2	4.8	6.1	9.3	13.8	30.0

change the value of γ_{bas} only in the "control (DQ) group": in practice, we are assuming that participants evaluate differently the baseline item if asked directly vs. in the CM. For instance, if the difference is .4, that implies that $\gamma_{bas} = -1$ in the CM group, but it equals -.6 in the control group. Or, if the difference is 0, that implies that $\gamma_{bas} = -1$ in both control and CM group. We only examine the case $n_{CM} = 1000$. Codes for replication are included in the .m file *MC3_Invariance.m.*

B.4 Fourth MC simulation: violating the assumption of statistical independence

In the final MC simulation the data generating process is as in the first set of MC simulations. We assume reliability of either .7 or .8 ($\bar{\alpha} \in \{.05, .34\}$)) and $\gamma_{bas} = -1$. We then explore whether we can detect violations of independence. First, we consider equation (8) with the following model parameters:

$$\mathbb{P}(U_i = 1|\theta_i, X_i) = \Phi(-1 + \beta_\theta \theta_i) \tag{1}$$

where $\beta_\theta \in \{-.5, -.25, -.1, 0, .1, .25, .5\}$. We then estimate models CM3a and CM4a, assuming that the baseline and target items are statistically independent (hence, we do not estimate the regression model in equation (8)). We use both single group (simulation file: *MC4_dep_PPC*) and 2-groups design (simulation file: *MC4_dep_grps_PPC*). We only examine the case $n_{CM} = 1000$. The results (reported in the main manuscript) show that the PPC of the simulations with 2-groups design is sensitive to violations of statistical independence. For the final set of simulations, we simulate data using the following model parameters for equation (8):

$$\mathbb{P}(U_i = 1|\theta_i, X_i) = \Phi(-1 + .4\theta_i) \tag{2}$$

$$\mathbb{P}(U_i = 1|\theta_i, X_i) = \Phi(-1 + .4\theta_i + .4x_i) \tag{3}$$

with $x_i \sim \mathcal{N}(0, 1)$. We then study whether we can correctly estimate the model parameters using again models CM3a and CM4a, with both single group (simulation file: *MC4_dep_pars*) and 2-groups design (simulation file: *MC4_dep_grps_pars*).

Figure 1: Estimation of prevalence of target item with models CM3a and CM3b ($N = 500$)

(a) Model CM3a (non-informative prior: $\log(\alpha_{bas}) \sim \text{Uniform}(0, 4)$)

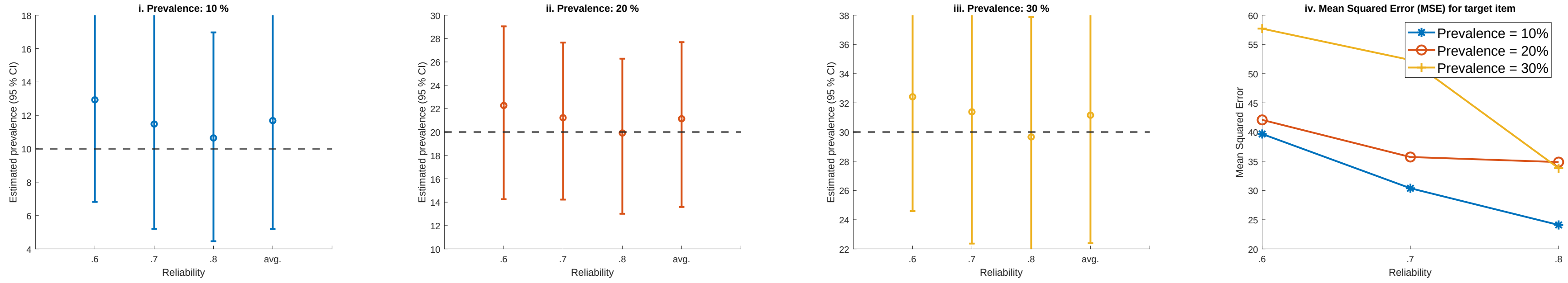

(b) Model CM3a (informative prior: $\log(\alpha_{bas}) \sim \log\mathcal{N}(\bar{\alpha}, .1)$)

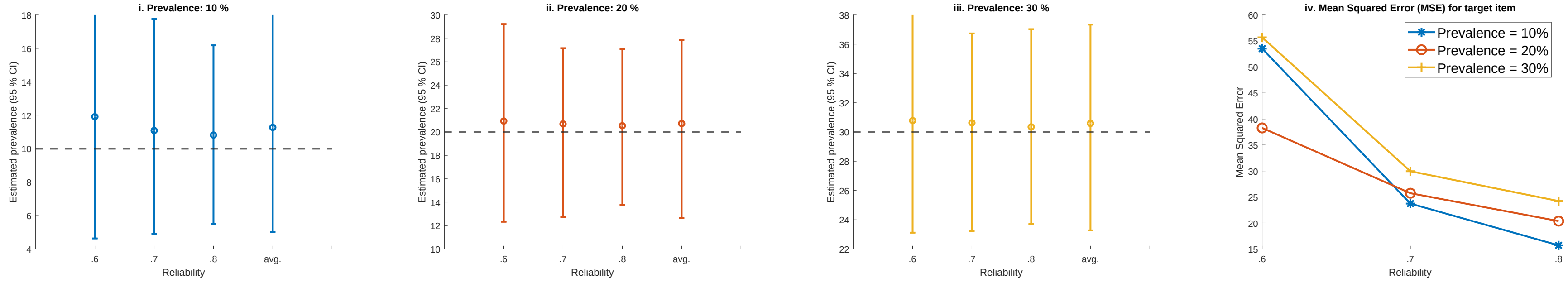

(c) Model CM3b

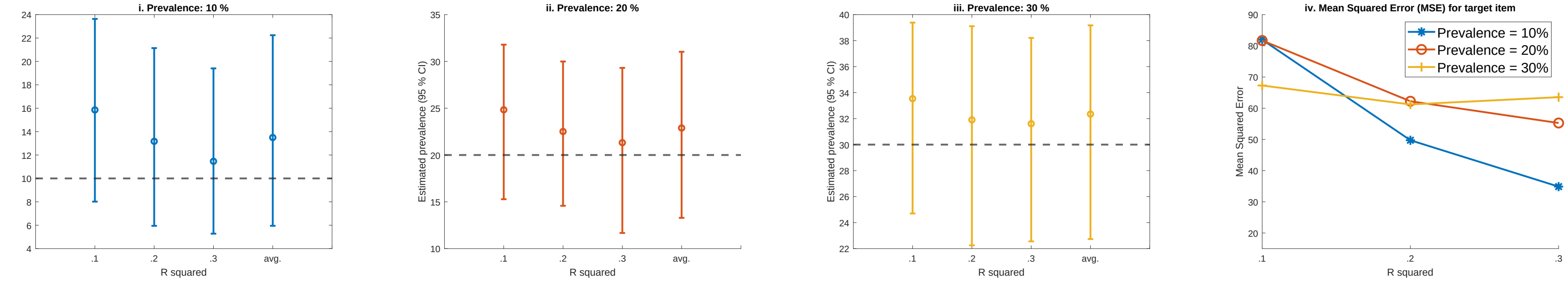

Figure 2: Estimation of prevalence of target item with models CM3a and CM3b ($N = 1000$)

(a) Model CM3a (non-informative prior: $\log(\alpha_{bas}) \sim \text{Uniform}(0, 4)$)

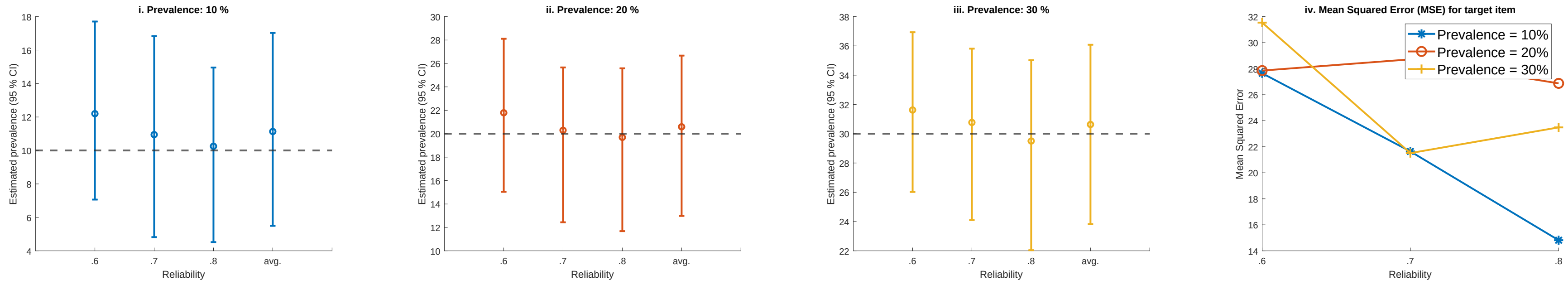

(b) Model CM3a (informative prior: $\log(\alpha_{bas}) \sim \log\mathcal{N}(\bar{\alpha}, .1)$)

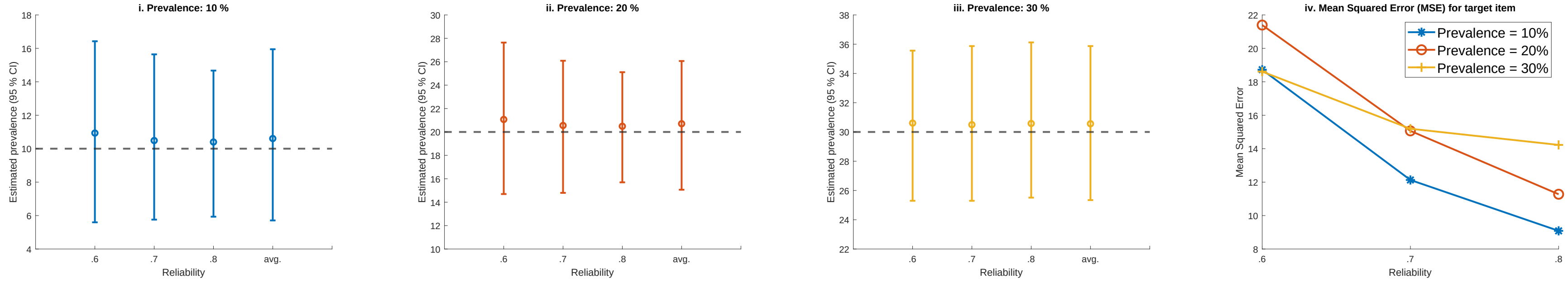

(c) Model CM3b

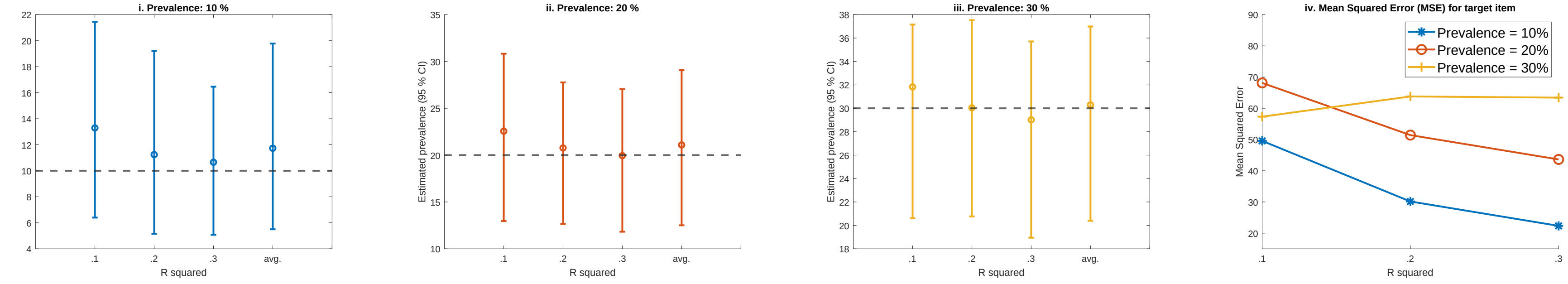

Figure 3: Estimation of prevalence of target item with models CM3a and CM3b ($N = 1500$)

(a) Model CM3a (non-informative prior: $\log(\alpha_{bas}) \sim \text{Uniform}(0, 4)$)

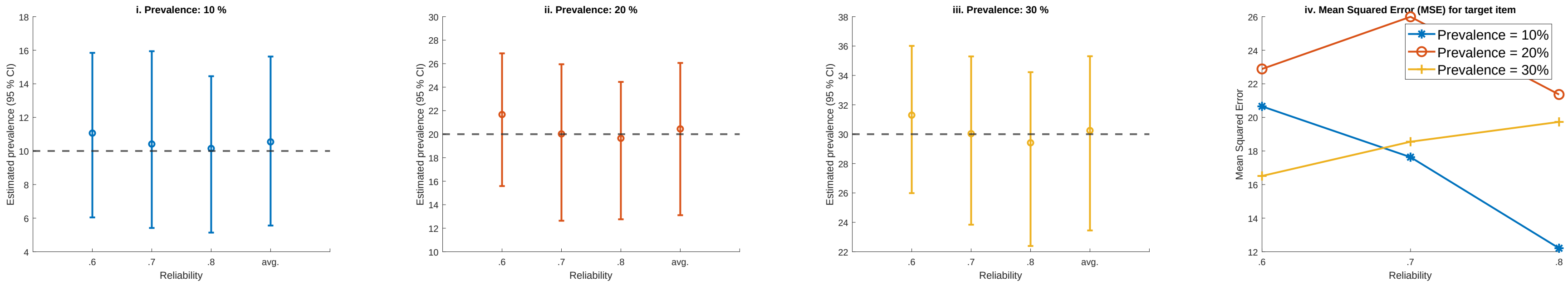

(b) Model CM3a (informative prior: $\log(\alpha_{bas}) \sim \log \mathcal{N}(\bar{\alpha}, .1)$)

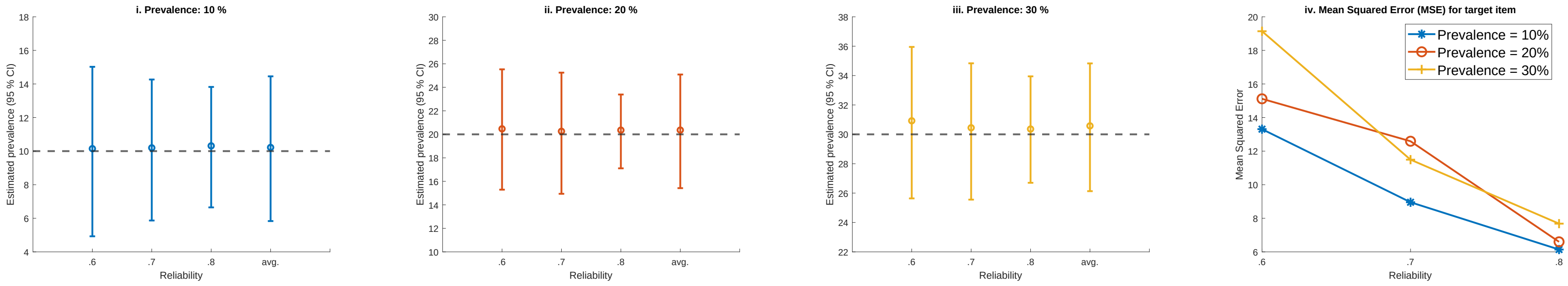

(c) Model CM3b

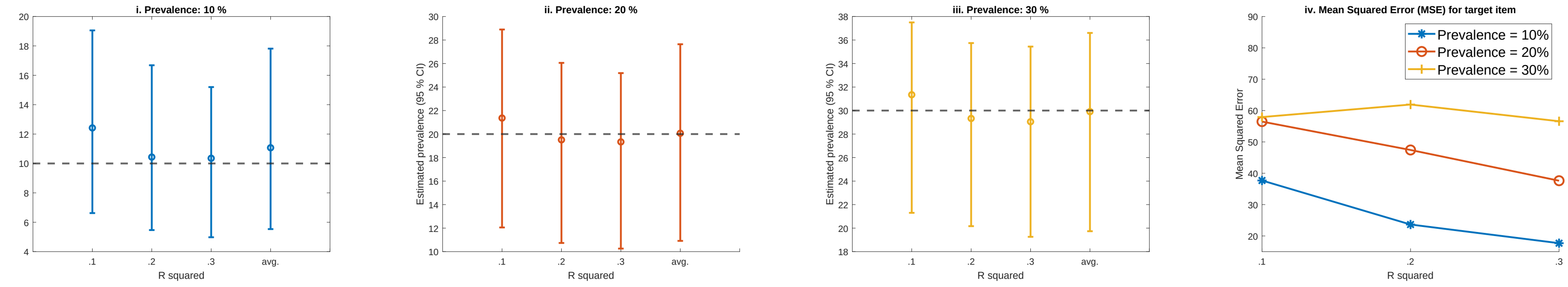

C Matlab and Python Codes to Implement CM

Codes and interactive app to replicate the results are available at the following OSF repository link: https://osf.io/wprtf/?view_only=c1584740c1b145b9a172b3a82e5ee775.

The Matlab codes replicate exactly the analyses reported in this paper. For the empirical analyses, we used 25000 burnin draws and 25000 draws to compute posterior statistics. To replicate the analyses for each statistical model (CM2, CM3a and CM4a) run the EmpStudy.m file on Matlab. Some illustrative code is provided and explained below. The dataset for the study (EmpStudy.xlsx) is included in the repository under "Data and Materials". Before running the estimation method, select the target item of interest, by inputing it as "Qs":

- "ZIP": The ZIP code of my home address begins with 6.
- "Wristwatch": I am wearing a wristwatch right now.
- "Verizon": I am a Verizon customer
- "WorkTrans": I would have a problem working with a transsexual coworker
- "WorkLGB": I would have a problem working with an openly lesbian, gay, or bisexual coworker
- "LGBprct": I am an LGBT individual

If the analysis should include the covariates to predict the target items, set CovOn = 1. If the analysis should focus on one of the two conditions ("Instructions" or "No Instructions") set Group = 1 and select the group of interest (GroupSet = "No Instructions"/"Instructions"). This will stratify the CM across the groups of interest. The Matlab file will load and clean the dataset. The final output includes average posterior prevalence of the target item (for both the CM and DQ groups), fit measures and (if needed) coefficients of the covariates to predict the target item for all three models. Some illustrative code, summarizing the important steps and the required inputs for each function, is provided below.

```
% Estimate models CM2, CM3 and CM4a in the empirical study
% set burnin, thinning, and total number of draws
burnin = 25000;
thin = 1;
mm = 25000;

Qs = 'Verizon';                          % select target question
 % if Group = 1, use instruction/no instruction condition
Group = 0;
GroupSet = 'No Instructions';        % select group
CovOn = 0;                               % predict target item
mData = readtable('EmpStudy.xlsx');   % load data

%% estimate models
% Model CM2 - output:
% 1.PostStats2: estimated probability of affirming target item with CM
    (model CM2), DQ and difference
% 2.FitMeasures2: posterior predictive p-value, DIC, and WAIC of Model
% 3.CoeffTable2: summary regression coefficients with CM and DQ
% 4.conv_diag2: convergence diagnostics
% 5.target_draws2: draws for target item prevalence
%
% input:
% 1. ListCM: list of responses to CM (1="response is different")
% 2. BasDQ:  responses to baseline question (as estimated in control
    group)
% 3. ListDQ: list of responses answered directly
% 4. mX:     optional, insert 0 if no covariates
%            matrix of independent variables (CM group)
```

```
% 5. mXdq:    matrix of independent variables (DQ group)
% 6. burnin: number of burnin draws
% 7. mm:      number of draws used to compute posterior statistics
% 8. thin:    thinning
% 9. StartValue: starting value of for prevalence of target item
[PostStats2, FitMeasures2, CoeffTable2, conv_diag2, target_draws2] ...
    = ModelM2(ListCM, BasDQ, ListDQ, mX, mXdq, ...
    burnin, mm, thin, StartValue);

% Model CM3a - output:
% 1. PostStats3a: estimated probability of affirming target item with CM (model CM3a), DQ and difference
% 2. FitMeasures3a: posterior predictive p-value, DIC, and WAIC of Model
% 3. ItsStats3a: summary baseline items statistics
% 4. CoeffTable3a: summary regression coefficients with CM and DQ
% 5. conv_diag3a: convergence diagnostics
% input:
% 1. ListCM: list of responses to Crosswise Model ("response is different")
% 2. Ctr:  Outside-the-CM items
% 3. ListDQ: list of responses answered directly
% 4. mX:      optional, insert 0 if no covariates
%             matrix of independent variables (CM group)
% 5. mXdq:    matrix of independent variables (DQ group)
% 6. PersPred: 1 if using also outside-the-CM items to predict target trait
% 7. burnin: number of burnin draws
% 8. mm:      number of draws used to compute posterior statistics
```

```
% 9. thin:    thinning
% 10. PriorA: vector of informative lognormal prior parameters (mean
   and
%              variance) for discrimination parameter, set 0 if using
%              uniform(0,4) prior
% 11. StartValue: starting value of for prevalence of target item
[PostStats3a, FitMeasures3a, ItsStats3a, CoeffTable3a, conv_diag3a] ...
      = ModelM3a(ListCM, Ctr, ListDQ, mX, mXdq, ...
      PersPred, burnin, mm, thin, PriorA, StartValue)

% Model CM4a - output:
% 1. PostStats4a: estimated probability of affirming target item with
   CM (model CM4a), DQ and difference
% 2. FitMeasures4a: posterior predictive p-value, DIC, and WAIC of
   Model
% 3. ItsStats4a: summary baseline items statistics
% 4. CoeffTable4a: summary regression coefficients with CM and DQ
% 5. conv_diag4a: convergence diagnostics
%
% input:
% 1. ListCM: list of responses to Crosswise Model ("response is
   different")
% 2. Ctr:  Outside-the-CM items
% 3. ListDQ: list of responses answered directly
% 4. BasDQ:  responses to baseline question (as estimated in control
   group)
% 5. mX:      optional, insert 0 if no covariates
%             matrix of independent variables (CM group)
% 6. mXdq:    matrix of independent variables (DQ group)
```

```
% 7. PersPred: 1 if using also outside-the-CM items to predict target
    trait
% 8. burnin: number of burnin draws
% 9. mm:     number of draws used to compute posterior statistics
% 10. thin:   thinning
% 11. StartValue: starting value of for prevalence of target item
[PostStats4a, FitMeasures4a, ItsStats4a, CoeffTable4a, conv_diag4a] ...
    = ModelM4a(ListCM, Ctr, ListDQ, BasDQ, mX, mXdq, ...
    burnin, mm, thin, StartValue);
```

Python codes are also available for estimating each statistical model (without comparison with the DQ group). In the example in the repository, the EmpStudy.py loads and cleans the data, and estimate each statistical model for the target item "I would have a problem working with a colleague who is transsexual". Some illustrative code to estimate model CM2 and model CM3a in Python is given below.

```
#!/usr/bin/env python3
# -*- coding: utf-8 -*-
"""
Created on Wed Jul 21 22:42:17 2021
"""

import pandas as pd
import numpy as np
from Routines import model_2
from Routines import model_3

# select burnin and number of draws
burnin = 1000
mm = 1000

##### import and clean data

# Model CM2 - output
# 1.post_stats_2: estimated prob. of affirming target item with CM (
    Model CM2)
# 2.fit_measures_2: posterior predictive p-value, DIC, and WAIC of
    Model
# 3.coeff_table_2: summary regression coefficients with CM and DQ
#
```

```
# input:
# 1. list_cm: list of responses to CM (1="response is different")
# 2. bas_dq:  responses to baseline question (from control group)
# 3. mX:      optional, insert 0 if no covariates
#             matrix of independent variables (CM group)
# 4. burnin: number of burnin draws
# 5. mm:      number of draws used to compute posterior statistics

post_stats_2, fit_measures_2, coeff_table_2 = model_2(list_cm, bas_dq, 0, burnin, mm)

# Model CM3 - output
# 1.post_stats_3: estimated prob. of affirming target item with CM (Model CM3)
# 2.its_stats_3: summary baseline items statistics
# 3.fit_measures_3: posterior predictive p-value, DIC, and WAIC of Model
# 4.coeff_table_3: summary regression coefficients with CM and DQ
#
# input:
# 1. list_cm: list of responses to CM (1="response is different")
# 2. out_cm:  Outside-the-CM items
# 3. mX:      optional, insert 0 if no covariates
#             matrix of independent variables (CM group)
# 4. burnin: number of burnin draws
# 5. mm:      number of draws used to compute posterior statistics

post_stats_3, its_stats_3, fit_measures_3, coeff_table_3 = model_3(list_cm, out_cm, 0, burnin, mm)
```

D Using the Crosswise Stand-alone Application

The following tutorial explains how to use the interactive app to estimate model CM2 and model CM3a as an example. We will provide guidelines for estimating models CM2 and CM3a.

Installation

Download the relevant files from the ”App_Windows” folder.

- If you do not have Matlab or the Matlab Runtime installed, to install the app download CrossWiserRuntime.app and follow the instructions for installation
- If you have Matlab or the Runtime installed already, to install the app download CrossWiser.app and follow the instructions for installation.

After having installed and started the app “CrossWiser”, this interface will be shown:

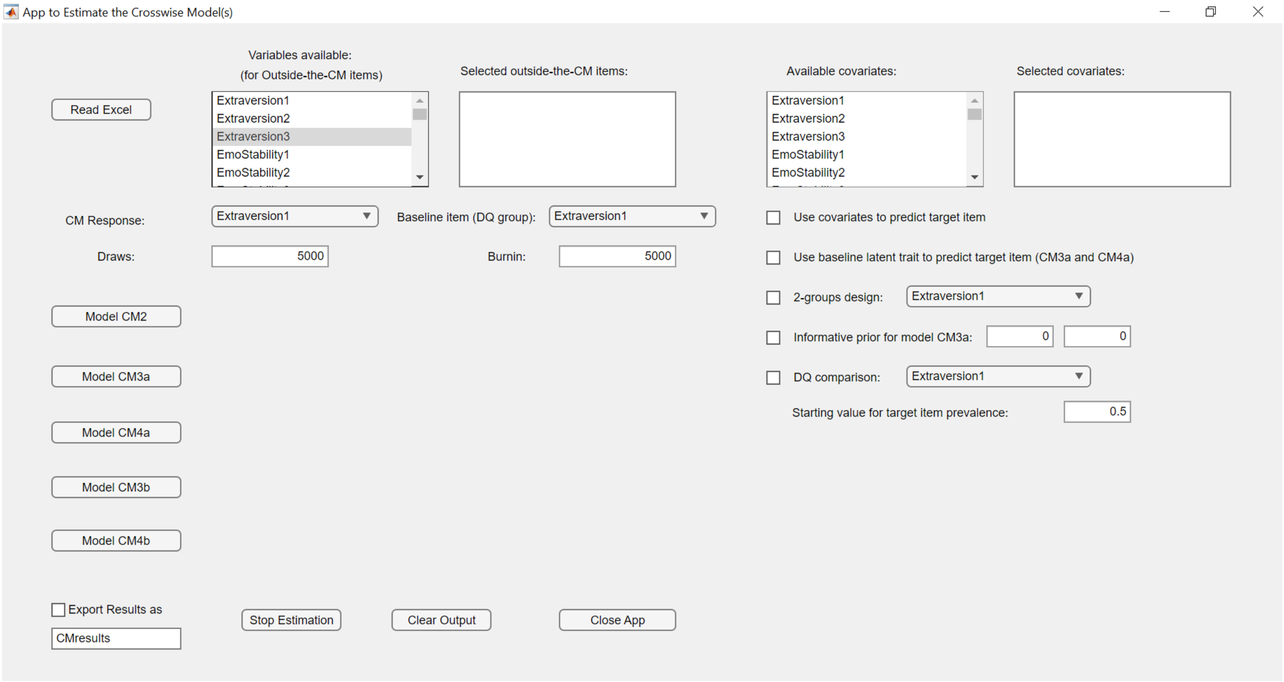

The app automatically uploads the dataset used in the empirical application. Click on Read Excel to load an alternative .xlsx file.

Estimating model CM2

1. Set the variable “WorkTransCM” as “CM response” (the variable must be coded as 1 for the option “my responses are different” and 0 otherwise)
2. Set the variable “WorkTransBas” as “Baseline item (DQ group)” (the variable must be coded as 1 for the option “yes” and 0 for the option “no”)
3. Set the number of draws and burnin: the default number of burnin draws is 5K and the default number of draws used to compute posterior statistics is 5K. If this is your first time using the app, we recommend using a low amount of draws (such as 250 for burnin and posterior draws)
4. If you want to export your results in .xlsx file, click on “Export Results as...” and then write the name of the .xlsx file (default is “CMresults.xlsx”). The excel file will be saved in the directory where the app is installed. Upon exporting the results, also a convergence plot will be automatically exported.
5. Click on Model CM2. While the estimation is running, a waitbar will pop up showing estimation progress

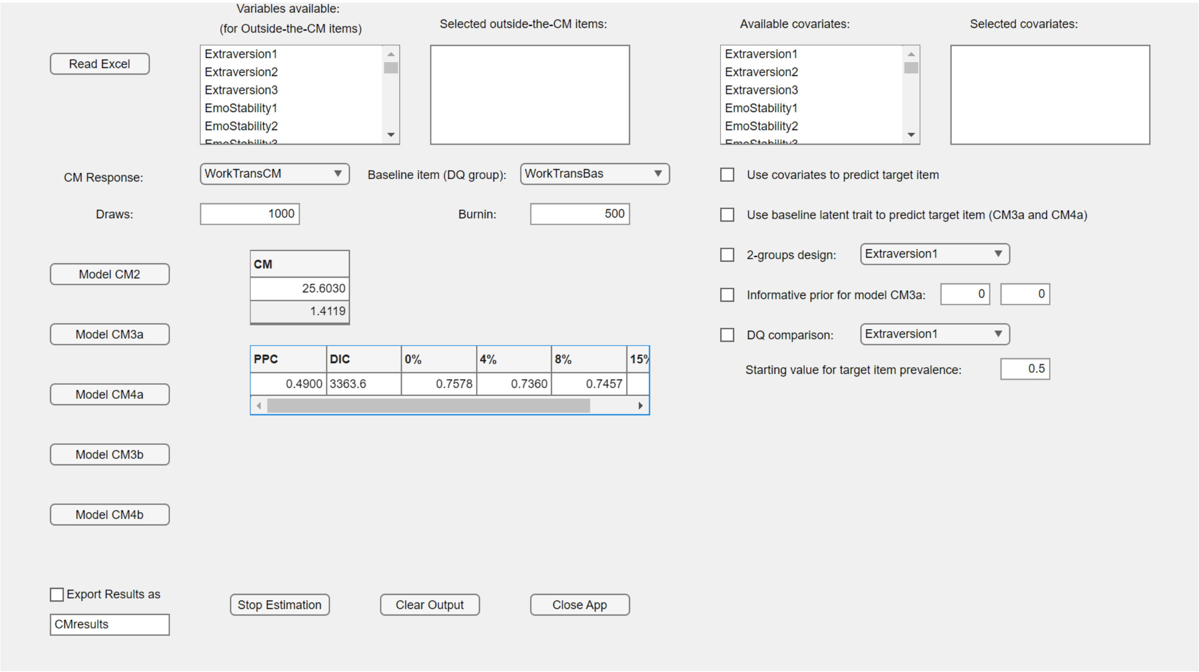

The output is the average response to the target item (25.6 in the example above), its standard deviation, measures of fit (p-value and DIC), and Geweke convergence diagnostic checks (x %, with $x = \{0, 4, 8, 15\}$ corresponds to a certain taper for autocorrelation in the posterior draws. Ideally one wishes all Geweke convergence outcomes to be above .05).

- If you want to stop the estimation, click the bottom button "stop estimation"
- If you want to clear the output, click on "clear output"
- If you want to close the app, click on "Close App"

Including covariates to predict the target item

If you want to include covariates to predict the target item, include these by selecting them on the top right box ("Variables available") - the selected covariates will be shown in the box on the right (Variables available). Then click on "Use covariates to predict target Item" and click on "Model CM2". Note: to select the items in the boxes "Variables available", *hold down* the "Ctrl"-key key and click on the items

If you want to change the variables selected, select them anew from the box: previously selected variables will be automatically erased.

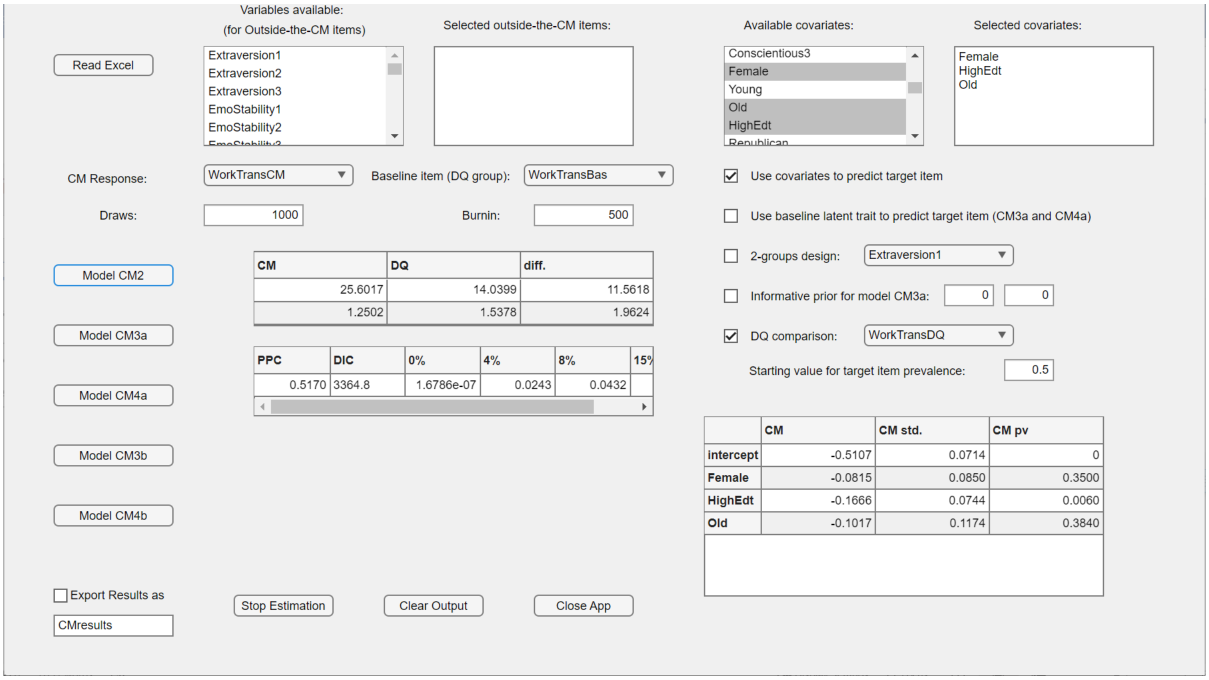

We can also compare the results from the CM model to some measures elicited directly using the box "DQ comparison". This is done with the variable "WorkTransDQ" in the example.

The output is the average response to the target item (25.6 in the example), its standard deviation, the results for DQ and comparison with the CM, measures of fit and convergence, and the coefficients for each variable selected in the Probit regression (showing coefficient, standard error and p-value).

Estimating Model CM3a

- Similarly to when selecting covariates in the earlier example, click on the variables "Conscientious1", "2" and "3" under "Variables available" in the top left box. They should appear under "Selected outside-the-CM items".
- Set the variable "WorkTransCM" as "CM response"
- Set number of draws and burnin
- If you want to export your results in .xlsx file, tick on "Export Results as..." and then write the name of the .xlsx file (default is CMresults)
- Click on Model CM3a

The output is the average response to the target item (27.1 in the example above), the measures of fit and the baseline item parameters (discrimination, difficulty and average probability of affirming the baseline item).

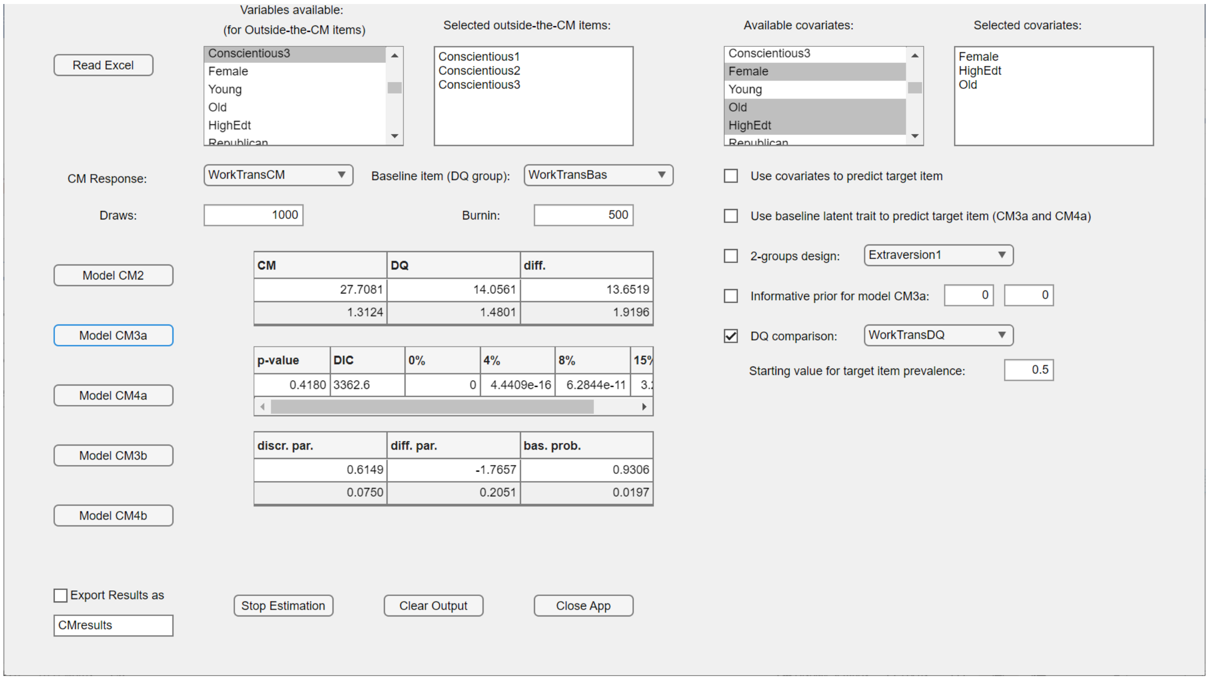

If you want to include covariates to predict the target item, follow the same steps as earlier.

E Suggested Items for Models CM3/CM4a

For future applications of the CM, we include examples of outside-the-CM items and baseline items that we employed in the current research. These items were used in our empirical application.

Component	Outside-the-CM items	Baseline items
**Extraversion**	I talk to a lot of different people at parties	I keep in the background
	I start conversations	I am quiet around strangers
	I am the life of the party	
**Emotional Stability**	I worry about things	I am relaxed most of the time
	I change my mood a lot	
	I get irritated easily	
**Agreeableness**	I feel little concern for others	I make people feel at ease
	I have a soft heart	
	I take time out for others	
**Conscientiousness**	I get chores done right away	I have a lot of self discipline
	I follow a schedule	I like order
	I leave my belongings around	

F Additional Empirical Analyses for Study 1

Correlation between target and baseline item in DQ

We report the empirical correlation between the binary target and baseline items in Table S7, as estimated in the control group. The results suggest that the significant correlations are due to chance: e.g. the largest correlation (9.1 %) is between wearing a wristwatch and making people feel at ease, with a high false discovery rate due to multiple hypotheses testing.

Subgroup analyses by instruction manipulation

In this section we examine the CM across various manipulations. In one condition, the CM was introduced with no detailed instructions. In the other condition, survey participants received de-

Nonsensitive Target Items	Baseline items	corr.	p-value	FDR
a. The ZIP code of my home address begins with 6	a. I keep in the background	-0.021	0.634	0.029
b. I am wearing a wristwatch right now	b. I make people feel at ease	0.091	0.036	0.008
c. I am a Verizon customer	c. I am relaxed most of the time	0.081	0.063	0.007
Sensitive Target Items				
e. I would have a problem working with a colleague who is LGB	e. I have a lot of self-discipline	0.009	0.828	0.031
d. I would have a problem working with a colleague who is transsexual	d. I like order	0.072	0.099	0.008
e. I am an LGBT individual	f. I am quiet around strangers	-0.029	0.508	0.029

Table S7: Empirical correlations between target and baseline items in the control group (n = 531). corr: Pearson correlation coefficient; FDR: False Discovery Rate

tailed instructions about the CM, an example and an explanation of why their privacy is protected. Exact instructions are included in the OSF repository. The results of the analyses when administering extensive instructions are given in Table S8 and without extensive instructions are given in Table S9. They are usually similar to the results of the main application, hence they are not discussed in detail. One notable exception of interest concerned the ZIP code item with model CM3a. Specifically, when implementing model CM3a (Table S9), in the “No Instructions” condition, 8.8 % of respondents affirm the item with CM. In contrast, in the “Instructions” condition, 16.7 % of respondents affirm the item with CM. Hence, the overall difference is driven the “Instructions” condition, perhaps because the instructions prime survey participants to the potential sensitivity of the ZIP code information.

Table S8: Attitudes towards LGBT people: estimates from models CM2, CM3a and CM4a (instructions condition)

Target Items	Model CM2		Model CM3a		Model CM4a		
	1. CM	2. CM-DQ	3. CM	4. CM-DQ	5. CM	6. CM-DQ	7. DQ
Non-sensitive target items							
a. The ZIP code of my home	16.1	9	16.7	9.6***	16.2	9***	7.1
address begins with 6	(4.6)	(4.7)	(2.4)	(2.6)	(1.9)	(2.2)	(1.1)
b. I am wearing a	29.1	6.6*	23.5	0.9	26.3	3.9	22.5
wristwatch right now	(2.3)	(2.9)	(3.3)	(3.7)	(2)	(2.7)	(1.8)
c. I am a Verizon customer	42.5	7.8*	34.1	-0.6	36.9	2.2	34.7
	(2.6)	(3.3)	(3.3)	(3.9)	(2.2)	(3)	(2.1)
Sensitive target items							
e. I would have a problem working	23.8	17.3***	27.1	20.6***	26.5	20***	6.6
with a colleague who is LGB	(3.1)	(3.3)	(2.8)	(3)	(2.1)	(2.3)	(1.1)
d. I would have a problem working	26.8	12.8***	27.9	13.8***	27.1	13.1***	14.1
with a colleague who is transsexual	(1.8)	(2.3)	(1.8)	(2.4)	(1.6)	(2.2)	(1.5)
e. I am an LGBT individual	21.3	10.4**	19.3	8.4***	20.1	9.3***	10.9
	(3.7)	(3.9)	(2.6)	(2.9)	(2)	(2.4)	(1.3)
Sample Size	N = 1876		N = 1345		N = 1876		N = 531

Table S9: Attitudes towards LGBT people: estimates from models CM2, CM3aand CM4a (no instructions condition)

Target Items	Model CM2		Model CM3a		Model CM4a		
	1. CM	2. CM-DQ	3. CM	4. CM-DQ	5. CM	6. CM-DQ	7. DQ
Non-sensitive target items							
a. The ZIP code of my home	23.2	16.1***	8.8	1.6	12.3	5.2*	7.1
address begins with 6	(4.13)	(4.3)	(3.4)	(3.6)	(2.1)	(2.4)	(1.1)
b. I am wearing a	21.9	-0.6	19.3	-3.2	19.6	-2.9	22.5
wristwatch right now	(2.4)	(3)	(3)	(3.5)	(2)	(2.7)	(1.8)
c. I am a Verizon customer	38.7	4	31.4	-3.3	33.2	-1.5	34.7
	(2.6)	(3.3)	(3.3)	(3.9)	(2.2)	(3)	(2.1)
Sensitive Target Questions							
e. I would have a problem working	22.5	16***	24.2	17.7***	23.8	17.3***	6.6
with a colleague who is LGB	(3.1)	(3.3)	(2.7)	(2.9)	(2)	(2.3)	(1.1)
d. I would have a problem working	24.5	10.4***	28.1	14.1***	26.4	12.3***	14.1
with a colleague who is transsexual	(1.8)	(2.3)	(1.6)	(2.2)	(1.5)	(2.1)	(1.5)
e. I am an LGBT individual	23.9	13***	19.2	8.4***	19.5	8.6***	10.9
	(3.5)	(3.8)	(2.3)	(2.7)	(1.9)	(2.3)	(1.3)
Sample Size	N = 1913		N = 1382		N = 1913		N = 531

Table S10: Model selection for full sample: PPC and DIC (instructions condition)

	Model CM2		Model CM3a		Model CM4a	
	1. PPC	2. DIC	3. PPC	4. DIC	5. PPC	6. DIC
Non-sensitive target items						
a. The ZIP code of my home address begins with 6	0.49	1758.3	0.46	1583	0.18	1581.7
b. I am wearing a wristwatch right now	0.49	1766.3	0.41	1715.4	0.85	1718.4
c. I am a Verizon customer	0.5	1858	0.43	1825.9	0.98	1830
Sensitive Target Questions						
e. I would have a problem working with a colleague who is LGB	0.49	1761.8	0.37	1733	0.29	1731.2
d. I would have a problem working with a colleague who is transsexual	0.49	1680	0.43	1670.4	0.47	1668.6
e. I am an LGBT individual	0.5	1766.3	0.36	1653	0.24	1651

Table S11: Model selection for full sample: PPC and DIC (no instructions condition)

	Model CM2		Model CM3a		Model CM4a	
	1. PPC	2. DIC	3. PPC	4. DIC	5. PPC	6. DIC
Non-sensitive target items						
a. The ZIP code of my home address begins with 6	0.49	1848.7	0.42	1597.5	0.63	1596.6
b. I am wearing a wristwatch right now	0.49	1728.2	0.45	1661.8	0.73	1661.6
c. I am a Verizon customer	0.5	1897.9	0.52	1852.2	0.97	1856.1
Sensitive Target Questions						
e. I would have a problem working with a colleague who is LGB	0.49	1799.2	0.45	1742.3	0.56	1741.4
d. I would have a problem working with a colleague who is transsexual	0.49	1684.3	0.43	1685.3	0.28	1688.5
e. I am an LGBT individual	0.5	1833.4	0.43	1690.5	0.39	1688.3

G Overview of past studies

We finally provide an overview of past studies, based on Sagoe et al. (2021). The overview gives sample sizes, and type and prevalence of baseline items used in previous research.

Table S23

	n (CM)	n (DQ)	Prevalence Baseline item	Baseline item
Atsusaka 2020	188	282	0.086	NA
	270	282	0.086	NA
	189	282	0.086	NA
	270	282	0.25	NA
	192	282	0.25	NA
	267	282	0.25	NA
Banayejeddi 2019	1740	440	0.083	Take one of your friends or relatives whose birthday you remember. Is his (her) birthday in March?
	1740	440	0.1	Take one of your friends or relatives whose birthday you remember. Is his (her) birthday between the 1st and 3rd of the month?
	1740	440	0.1	Take one of the numbers 0 to 9 and do not change it. Is the number 8 your choice?
	1740	440	0.1	Take the phone number of your friends or relatives you know and do not change that. Is the first digit of the phone number 4?
	1740	440	0.1	Take the cell phone number of one of your friends or relatives you know and do not change that. Is the last digit of the cell phone number 6?
	1740	440	0.125	Take one letter of the groups of eight letters from the Persian alphabet and do not change that. Does the selected letter belong to the second group?
Canan 2021	313	252	0.235	Was your mother born in February, April or November? If you do not know your mother's birthday, think of your grandmother or some other woman whose birthday you know
Coutts 2011	310	96	0.25	Is your mother's birthday in January, February or March?
	310	96	0.25	Is your father's birthday in October, November or December
Eslami 2013	2993		0.25	Were you born in spring?
Gingerich 2015	4200	4200	0.25	My mother was born in October, November, or December
Heck 2018	322		0.25	Is your mother's birthday between May and July?
	322		0.25	Is your father's birthday between August and April?
Hoffmann 2015	526	138	0.158	I was born in November or December
Hoffmann 2016	1312	1312	0.158	My father was born in November or December
	1312	1312	0.158	
Hoffmann 2017	401	401	0.17	I was born in November or December
Hoffmann 2020	454	466	0.158	My father was born in November or December
	465	452	0.158	My mother was born in November or December
Höglinger 2016	1008	1004	0.25	Is your mother's birthday in January, February, or March

	n (CM)	n (DQ)	Prevalence Baseline item	Baseline item
	1008	1004	0.25	Is your father's birthday in October, November, or December?
	1008	1004	0.25	
	1008	1004	0.25	
	1008	1004	0.25	
Höglinger 2017	1123	562	0.25	Is your mother's birthday in January, February, or March?
	1123	562	0.25	
	1123	562	0.25	
	1123	562	0.25	
	1123	562	0.25	
Höglinger 2018	1168	387	0.25	Is your father's birthday in October, November, or December?
	1145	382	0.25	
	2306	768	0.25	
	2313	768	0.25	
	2310	768	0.25	
Hopp 2019	144	144	0.333	Does your matriculation number end in 1, 2, or 3?
	144	144	0.24816	Does your birthday fall in the first quarter of the year?
Jensen 2020	523	536	0.158	Is your father's birthday in January or February? (if you don't know please use the birthday of another family member or a good friend)
	523	536	0.158	your mother's birthday in January or February? (if you don't know please use the birthday of another family member or a good friend)
Jann 2012	358	116	0.25	Is your mother's birthday in January, February, or March?
	358	116	0.25	Is your father's birthday in October, November, or December?
Jerke 2021	3008	3012	0.3325	In the last 12 months, I have attended more than four conferences
	1792	1765	0.2926	In the last 12 months, I've worked on at least one research proposal
Johann 2017	1205	1205	0.25	Is your mother's birthday in January, February, or March?
Kazemzadeh 2016	553		NA	
Khosravi 2015	1644		NA	Think of the password of one of your ATM cards, which you use more often. Is the final figure of this password one of the numbers 5, 6, or 7?
	1644		NA	the final figure of your ID number 2, 4, or 6? Were you born in summer? Think of the number of your father's or an acquaintance's house and do not change it. Is the rightmost figure 2, 4, or 6?
	1644		NA	Think of a friend or acquaintance whom you know very well and know when exactly he was born. Is his or her birthday between the first and the tenth day of the month?

	n (CM)	n (DQ)	Prevalence Baseline item	Baseline item
	1644		NA	Think of one of your friends that you know his/her mobile number. Is the rightmost figure of his/her mobile number 2, 4 or 6?
Klimas 2019	224	224	0.167	Is your mother's birthday in January or February
Korndörfer 2014	862	305	0.2471	Is your mother's birthday in January, February, or March?
Kundt 2014	256	137	0.141	Is the first digit of your of your friend's house-number 7, 8, or 9?
Kundt 2017	422		0.2	Is the last digit of your best friends phone number/of the number of the person you call most often 0 or 1?
Lacker 2020	253	253	0.166	Whether the respondents' mother was born in January or February
Lehrer 2019	867	2597	0.3	We asked you to think of a friend or relative whose house number is known to you. Is that house number's firstdigita1,2,3ora4?
Meisters 2020a	2136	577	0.158	I was born in November or December
Meisters 2020b	911	450	0.158	My father was born in November or December/My father was born between January and October
Mieth 2021	943	491	0.242	Is your mother's birthday in May, June or July?
Mirzazadeh 2018	265	265	0.1	Pick a card with yes/no
Nakhaee 2013	298	298	0.25	Is your birth in spring?
Nasirian 2018	128	1090	NA	Was he/she born in a special solar month?
	128	1090	NA	Is the number of the main family members four?
	128	1090	NA	Do his/her main family members own vehicles (car, motorcycle)?
	128	1090	NA	Do his/her main family members own vehicles (car, motorcycle)?
Oliveros 2019	4193	4193	0.264	My mother was born in October, November, or December
Özgül 2020	534	178	0.2471	Is your mother's birthday in January, February, or Not applicable March?
Roberts 2014	98	93	0.247	Is your birthday in January, March or April?
	98	93	0.249	Is your birthday in August, November or December?
	98	93	0.250	Is your birthday in January, April or September?
	98	93	0.252	Is your birthday in June, August or December?
	98	93	0.241	Is your birthday in February, June or November?
Safiri 2019	1730	1730	0.300	Think of your parents' or someone else's house number and do not change it. Is the left side digit 5, 6, or 7?
	1730	1730	0.250	Please think of a friend whose birth date you know, and do not change it. Is his/her birthday in spring?
	1730	1730	0.30	Think of the pin number of one of your ATM cards that you use more often: is the left side digit 5, 7, or 8?

	n (CM)	n (DQ)	Prevalence Baseline item	Baseline item
	1730	1730	0.3	Think of the ID number of a friend or acquaintance: is the right side digit 6, 7, or 8?
	1730	1730	0.329	Please think of the birth date of you're a friend or acquaintance whose birth date you know precisely and do not change it: is the day between the first and tenth of a month?
	1730	1730	0.3	Please think of a friend or acquaintance whose mobile number you know and do not change it: is the right side digit of the number 7, 8, or 9?
Schnapp 2019	103		0.167	Was your mother born in January or February?
	103		0.167	Was your father born in January or February?
	103		0.088	Please think of your main residence. Is the first digit of your house number 8 or 9?
Shamsipour 2014	1490	1568	0.3285	Take one of your friends or acquaintances whose birthday you remember. Is their birthday between the 1st and10th of the month?
	1490	1568	0.3	Take the pin code of one of your ATM cards that you use frequently. Is the last digit 5, 6, or 7?
	1490	1568	0.3	Take one of your friends or acquaintances whose cell phone number you know by heart. Is the last digit 1, 2, or 3?
	1490	1568	0.3	Take the street number of your parents or one of your acquaintances, and do not change it. Is the last digit of this number 2, 4, or 6?
Vakilian 2014	100		NA	Do you have any friend or relative named Ali or Mohammad?
Vakilian 2016	1500		0.25	Consider one of your friends or acquaintances that you know his/her cell phone number, is the last digit of his/her phone one of the figures 1–3?
Vakilian 2019	1500		0.24	Please recall the cell phone number of one of your friends or acquaintances. Is the last digit 1, 2 or 3?
	1500		0.24	Please think about one of your female friends or acquaintances, is her name Fateme, Zahra, or Maryam (either singularly or in combination with other names)?
	1500		0.24	Please recall the number of your fathers (or one of your acquaintances) house. Is the right digit 2, 4 or 6?
Walzenbach 2019	855	485	0.25	Is your father's birthday in January, February or March?
Waubert de Puiseau 2017	1104	1140	0.158	I was born in November or December
Median:	1008	Avg.:	0.25	

References

De Jong, M. G., & Pieters, R. (2019). Assessing sensitive consumer behavior using the item count response technique. *Journal of Marketing Research*, *56*(3), 345–360.

Fox, J.-P. (2010). *Bayesian item response modeling: Theory and applications*. Springer Science & Business Media.

Gregori, M. (2022). Codes and app to estimate crosswise models [Retrieved from: https://osf.io/wprtf/?view_only=c1584740c1b145b9a172b3a82e5ee775].

Sagoe, D., Cruyff, M., Spendiff, O., Chegeni, R., De Hon, O., Saugy, M., van der Heijden, P. G., & Petróczi, A. (2021). Functionality of the crosswise model for assessing sensitive or transgressive behavior: A systematic review and meta-analysis. *Frontiers in Psychology*, *12*.
